# Peripheral signaling pathways contributing to non-histaminergic itch in humans

**DOI:** 10.1186/s12967-023-04698-z

**Published:** 2023-12-12

**Authors:** Andrea Fiebig, Victoria Leibl, David Oostendorf, Saskia Lukaschek, Jens Frömbgen, Maral Masoudi, Andreas E. Kremer, Marion Strupf, Peter Reeh, Miriam Düll, Barbara Namer

**Affiliations:** 1 Research Group Neuroscience, Interdisciplinary Centre for Clinical Research, Faculty of Medicine, RWTH Aachen University, Aachen, Germany; 2 Institute of Neurophysiology, Uniklinik RWTH Aachen University, Aachen, Germany; 3 Institute of Physiology and Pathophysiology, University of Erlangen-Nürnberg, 91054 Erlangen, Germany; 4 Department of Gastroenterology and Hepatology, University Hospital Zürich, University of Zürich, Zurich, Switzerland; 5 Department of Medicine 1, University Hospital Erlangen and Friedrich-Alexander-University Erlangen-Nürnberg, Erlangen, Germany

**Keywords:** Microneurography, β-Alanine, BAM 8-22, Cowhage, Discharge patterns, Spatial contrast, Single-fiber recording, Nociceptor, Pruriceptor

## Abstract

**Background:**

Chronic itch (chronic pruritus) is a major therapeutic challenge that remains poorly understood despite the extensive recent analysis of human pruriceptors. It is unclear how the peripheral nervous system differentiates the signaling of non-histaminergic itch and pain.

**Methods:**

Here we used psychophysical analysis and microneurography (single nerve fiber recordings) in healthy human volunteers to explore the distinct signaling mechanisms of itch, using the pruritogens β-alanine, BAM 8-22 and cowhage extract.

**Results:**

The mode of application (injection or focal application using inactivated cowhage spicules) influenced the itch/pain ratio in sensations induced by BAM 8-22 and cowhage but not β-alanine. We found that sensitizing pre-injections of prostaglandin E2 increased the pain component of BAM 8-22 but not the other pruritogens. A-fibers contributed only to itch induced by β-alanine. TRPV1 and TRPA1 were necessary for itch signaling induced by all three pruritogens. In single-fiber recordings, we found that BAM 8-22 and β-alanine injection activated nearly all CM-fibers (to different extents) but not CMi-fibers, whereas cowhage extract injection activated only 56% of CM-fibers but also 25% of CMi-fibers. A “slow bursting discharge pattern” was evoked in 25% of CM-fibers by β-alanine, in 35% by BAM 8-22, but in only 10% by cowhage extract.

**Conclusion:**

Our results indicate that no labeled line exists for these pruritogens in humans. A combination of different mechanisms, specific for each pruritogen, leads to itching sensations rather than pain. Notably, non-receptor-based mechanisms such as spatial contrast or discharge pattern coding seem to be important processes. These findings will facilitate the discovery of therapeutic targets for chronic pruritus, which are unlikely to be treated effectively by single receptor blockade.

**Supplementary Information:**

The online version contains supplementary material available at 10.1186/s12967-023-04698-z.

## Introduction

Itch and pain are two distinct unpleasant sensations that function as warnings and are therefore essential for human survival. However, these sensations can become chronic, massively reducing the quality of life for affected patients [1]. Chronic itch, also known as chronic pruritus, is a therapeutic challenge because it is currently unclear how itch and pain signaling are differentiated. Indeed, there is some overlap between these sensations, with some patients describing a “burning itch” or “itchy sting”. Nociceptive and pruriceptive stimuli also influence each other, and share common signaling pathways. For example, pain induced by scratching reduces itch, and information about both stimuli is conducted via the same anatomical pathways [2, 3]. In the periphery unmyelinated afferent nerve fibers, so called C-fibers, have been shown to be activated by chemical substances causing itch and pain in human and itch and pain indicative behavior in rodents [4]. It is still not clear, if myelinated A-fibers are activated by pruritogens in human and if they play a role especially in non-histaminergic itch [5]. Especially, mechanosensitive C-fibers in human are activated by nociceptive heat and are necessary to set the heat pain threshold in humans, but are also strongly activated by cowhage spicules, causing strong itch sensations. Thus, it seems that the same peripheral nerve fiber type can signal both, itch and pain. Apart from this fact, also tight interactions between itch and pain pathways in the spinal cord and brain exist.

Research on itch pathways has largely focused on animal models and most knowledge is derived from the identification of specific receptors and nerve fiber subclasses for pruritogens. However, the differentiation between itch and pain signaling in humans is still incompletely understood. Four theories have been proposed to explain itch signaling [6]. These are known as the *labeled line* (neuronal specificity), *spatial contrast*, *population coding* and *temporal discharge pattern* hypotheses [7].

The *labeled line* hypothesis proposes exclusive primary afferent pathways for pain and itch [3, 8]. In rodents, non-human primates and humans, histamine-sensitive C-nociceptors were identified as part of a specialized pruritic pathway, suggesting a labeled line for histaminergic itch [9–11]. Single nerve fiber recordings in humans (microneurography) revealed that histamine causes long-lasting activation correlating with the time course of itch sensations, but only in a specific subgroup of non-mechanosensitive (silent or sleeping) C-fibers. However, these fibers can also respond to noxious heat [4, 12]. In mice, different pruritogens activate specific subtypes of G protein-coupled receptors (GPCRs) involved in itch signaling: chloroquine activates MrgprA3, bovine adrenal medulla peptide 8-22 (BAM 8-22) activates MrgprC11, β-alanine activates MrgprD, and cowhage (mucunain) activates protease-activated receptors PAR2 and PAR4 [13–15]. These receptors presumably label neuron populations that signal itch sensations in mice. The selective activation of a specific neuron class carrying the MrgprA3 receptor by the classical algogen capsaicin results in pure itch (scratching) behavior in mice [13].

The hypothesis of *spatial contrast* to distinguish itch from pain is based on the concept that a pruritogen generates a sharp contrast between a few strongly activated nociceptors surrounded by many non-activated nerve endings innervating the affected skin area, whereas an algogen would consistently excite most nociceptors in the same area, including those responding to the itch stimulus [7, 16, 17]. This allows for specific pruriceptors dispersed among skin nociceptors, signaling itch if exclusively activated but contributing to pain if collectively activated with their neighbors. In agreement, the algogen capsaicin and the pruritogen lysophosphatidic acid (LPA) both cause itching when applied focally to the upper layers of the skin using inactivated cowhage spicules, whereas diffuse application by intracutaneous injection results in both agents causing pain [17, 18]. However, single versus co-application of two different pruritogens activating different GPCRs (MrgprD and MrgprX1) did not cause a switch from itch to pain in healthy human subjects [19], although this may reflect the presence of both Mrgpr receptors on the same human neuron populations [20].

Although no distinct discharge patterns were observed in C-fibers stimulated with the pruritogen histamine versus the algogen mustard oil [4, 21], non-histaminergic itch caused by cowhage in monkeys induced a specific discharge pattern characterized by short bursts [22]. The hypothesis of a *temporal discharge pattern* is supported by the finding that the same neuron population can cause either itch or pain behavior in mice when activated by ionotropic or metabotropic receptors, respectively [23]. Furthermore, temporal aspects of neuronal discharge influence the transmission of potential itch signals in the spinal cord [24].

Finally, the *population coding hypothesis* arises from our recent observation that LPA evokes either itching or pain sensations depending on the application mode. Intracutaneous injection leads to burning pain and the strong activation of CMi-fibers whereas focal and superficial application causes itching with predominantly CM-fiber activation and less CMi-fiber activation. No known substance causes pain solely by activating CM-fibers without also activating CMi-fibers, suggesting there may be population-level coding during a switch from itch to pain, when non-histaminergic CMi-fibers are co-activated.

Given our incomplete understanding of non-histaminergic itch signaling in humans [25], here we investigated the underlying mechanisms using psychophysical experiments and microneurography (single nerve fiber recordings) in human volunteers. We evaluated current theories using the three well-characterized pruritogens β-alanine, BAM 8-22 and cowhage with different application routes.

## Methods

### Subjects

Thirteen female and five male healthy volunteers (age 19–45 years) took part in the microneurography study. None of the participants suffered from any neurological, dermatological or other chronic medical condition, or took regular or acute medication 24 h prior to the experiments. Subjects were recruited at the medical faculty of the University of Erlangen and the University of Aachen by advertising in medical lectures and social media groups used by medical and dental students. The subjects were comprehensively informed about the experimental procedures and they gave their written informed consent according to the Declaration of Helsinki. The study was conducted at the Institute of Physiology, Medical Faculty, RWTH Aachen University and at the University of Erlangen-Nürnberg and approved by the local ethics committees.

### Substances and application

The pruritogens β-alanine (Cat. No. 146064, Sigma, Taufkirchen, Germany), BAM 8-22 (Cat. No. SML0729, Sigma, Taufkirchen, Germany), cowhage extract and chloroquine (Resochin, Bayer, Leverkusen, Germany) were either injected intracutaneously (50 µL) using a 0.3-mL 30 G insulin syringe (Becton–Dickinson, Le Pont de Claix Cedex, France) or focally applied via heat-inactivated cowhage spicules by inserting the spicules with tweezers into the skin. The spicules were soaked with 89 mg/mL β-alanine, 4 mg/mL BAM 8-22 or 50 mg/mL chloroquine before application. We used lower concentrations of 8.9 mg/mL β-alanine, 0.04 mg/mL BAM 8-22, 25 mg/mL chloroquine, and ~ 0.023 mg/mL cowhage extract for injection. We used synthetic interstitial fluid (SIF) as a diluent and control (107.8 mM NaCl, 3.5 mM KCl, 1.5 mM CaCl_2_, 0.7 mM MgSO_4_, 26.2 mM NaHCO_3_, 1.7 mM NaH_2_PO_4_, 9.6 mM sodium gluconate, 5.5 mM glucose, 7.7 mM sucrose, pH 7.4). In some experiments, we pre-injected subjects with 10^–6^ M (100 µL) prostaglandin E2 (PGE2, Cat. No. P5640, Sigma, Taufkirchen, Germany) or 10^–7^ M (100 µL) bradykinin (Cat. No. B3259, Sigma, Taufkirchen, Germany) or with 100 µL (10 μM) of the TRPA1 channel blocker A-967079 (Cat. No. 4716, Tocris, Wiesbaden-Nordenstadt, Germany) or 100 µL (1 μM) of the TRPV1 channel blocker BCTC (Cat. No. SML0355, Sigma, Taufkirchen, Germany) as previously validated [26].

To prepare the cowhage extract, we kneaded ~ 5 g of cowhage spicules in an autoclave bag before transfer to a conical flask containing 300 mL of extraction buffer (0.1 M NaCl, 1 mM l-cysteine, pH 5.6). The suspension was stirred at 900 rpm for 4 h at 4 °C before sterile filtration and concentration using a 30 kDa Amicon ultrafilter. The protein concentration in the ultrafiltrate was 2.25 mg/mL. This was diluted 1:100 with sterile SIF before injection. The extract was stored at − 21 °C.

### Psychophysical analysis

Five separate double-blinded experimental series were applied to different cohorts of subjects, who rated the intensity of itch and pain sensations verbally on a numerical rating scale (NRS) from zero to 10. Zero was defined as no sensation and 10 as the maximum imaginable pain or itch. A pain rating of 1 was defined as the minimal sensation the volunteer would cause pain in contrast to any other itchy, neutral, or pleasant sensation and as example the nociceptive sensation evoked by pulling on few body hairs was given. The scratch threshold was set at NRS 3. If the subjects felt a sensation that was subjectively neither considered painful nor itchy, they were instructed to rate this sensation as 0.5. Itch was defined as an unpleasant sensation that evokes a desire to scratch. The pain rating comprised different unpleasant sensations such as sticking, pricking or burning as long as they were classified as painful by the volunteers. Since we wanted to compare the itch/pain ratio we only ask for pain ratings and not for different nociceptor sensations (stick, prick and burn) as it was performed in previous publication [15]. In our pilot experiments we recorded itch and pain for longer time periods at least over 10 min. In those experiments after 6 min were only few volunteers who rated itch or pain ≥ 1. Therefore, we decided to restrict the time for the experiments included in the manuscript to 6 min. Superficial blood flow in the forearm was measured by laser Doppler imaging using a moorLDI2-VR 2001 device (Moor Instruments, Axminster, UK). The precise area of axon-reflex vasodilation was determined offline using MLDI 3.0 software (Moor Instruments).

#### Experiment 1: application modes of pruritogens

We enrolled 24 healthy subjects aged 20–48 years in experiment 1 (β-alanine 13 female, 11 male; BAM 8-22 14 female, 8 male; chloroquine 15 female, 9 male; cowhage 13 female, 11 male; control with SIF 13 female, 11 male). Microinjections (50 µL) or focal applications (~ 30 spicules) of β-alanine, BAM 22-8, chloroquine, cowhage extract and SIF were carried out double-blinded in a random order on the volar forearms at 10-min intervals. Verbal itch and pain ratings were obtained every 10 s for 6 min. Superficial blood flow was recorded within a skin area of 4.5 × 4.8 cm (130 × 137 pixels) by laser Doppler imaging at a distance of 30 cm. Skin blood flow baseline images were scanned before each application, the first image was taken after 1.5 min and the second image after 3.5 min. Some of the data were included in the doctoral thesis of VL and have been published as a monograph in German.

#### Experiment 2: selective A-fiber block

We enrolled 20 healthy subjects (15 female, five male) aged 19–34 years in three experimental sessions on different days. In each session, the pruritogen was injected into the autonomous innervation territory of the superficial radial nerve on the dorsal side of one hand during selective A-fiber pressure block, and in the other hand without block as previously described and validated [27, 28]. We used a weight of 1.2 kg attached to a well-padded sling (4.7 cm wide, 25 cm long) which was placed on the proximal wrist to exert light pressure on the superficial radial nerve [29]. The progress of the selective A-fiber block was evaluated every 5 min starting at 20 min after initiating the pressure block by testing cold and warm perception abilities using cold or warm metal bars and mechanical stimulation with OptiHair_2_-Set von Frey filaments (Marstock Nervtest, Heidelberg, Germany). When subjects could no longer sense the cold metal bar, but warm and sharp stimuli were still judged correctly, the selective A-fiber block was assumed effective. During the continued nerve block, 50 µL of β-alanine, BAM 8-22, chloroquine, or cowhage extract, each in a separate experimental session, was injected intracutaneously into the innervation area of the superficial radial nerve and verbal NRS ratings were obtained every 10 s for 5 min. Control experiments without nerve block were performed on the contralateral arm at corresponding skin sites.

#### Experiment 3: pharmacological blockade of TRPA1 and TRPV1

We enrolled 20 healthy subjects for BAM 8-22 injection (17 female, three male; mean age 23 years), 19 for β-alanine injection (17 female, three male; mean age 24 years) and 17 for cowhage extract injection (15 female, two male; mean age 24 years). The roles of TRPA1 and TRPV1 receptors in itch sensations evoked by β-alanine, BAM 8-22 and cowhage extract were determined by injecting 100 µL (10 μM) of the TRPA1 channel blocker A-967079, or 100 µL (1 μM) of the TRPV1 channel blocker BCTC, 2 min before injecting 50 µL of the pruritogen or control solution at the same site [26]. Verbal NRS ratings were obtained every 10 s for 5 min. As control experiments, SIF was pre-injected instead of the channel blockers on the contralateral arm at corresponding skin sites.

#### Experiment 4: interaction with inflammatory mediators

We enrolled 16 healthy subjects (13 female, three male) aged 19–30 to determine whether a larger pain component of the mixed itch/pain sensation can be caused by the pre-injection of an inflammatory mediator before the pruritogens β-alanine, BAM 8-22, or cowhage extract. First, 100 µL of PGE2 or SIF as a control solution was pre-injected superficially into the skin of one forearm. When the PGE2-induced sensations subsided, one of the pruritogens or SIF (50 µL) was applied to the same skin site and verbal NRS ratings were obtained every 10 s for 420 s.

#### Experiment 5: electrical stimulation

Electrical stimulation with 0.2-mA sine-wave pulses (4 Hz) for a duration of 60 s was induced using a Digitimer DS5 constant current stimulator (Digitimer, Welwyn Garden City, UK) and an NI USB-6221 pulse generator (National Instruments, Austin, TX, USA) controlled using a DAPSYS recording and stimulation system (BrianTurnquist, Bethel University, St. Paul, MN, USA). We examined 15 healthy subjects (seven male, eight female) aged 19–47 years. We used a pair of L-shaped blunted bipolar platinum–iridium surface electrodes (diameter 0.4 mm, distance 2 mm; Cephalon, Nørresundby, Denmark) [30]. We assessed itch and pain sensations during a 1-min sine wave stimulus before and after a 50-µL injection of SIF, 100 mM β-alanine or 0.04 mg/mL BAM 8-22, or a 30-µL injection of 0.023 mg/mL cowhage extract, or histamine iontophoresis (1% in distilled water). Itch and pain were rated verbally every 10 s during the 1-min electrical stimulation.

### Microneurography

#### Microneurography recordings

**Fig. 1:**
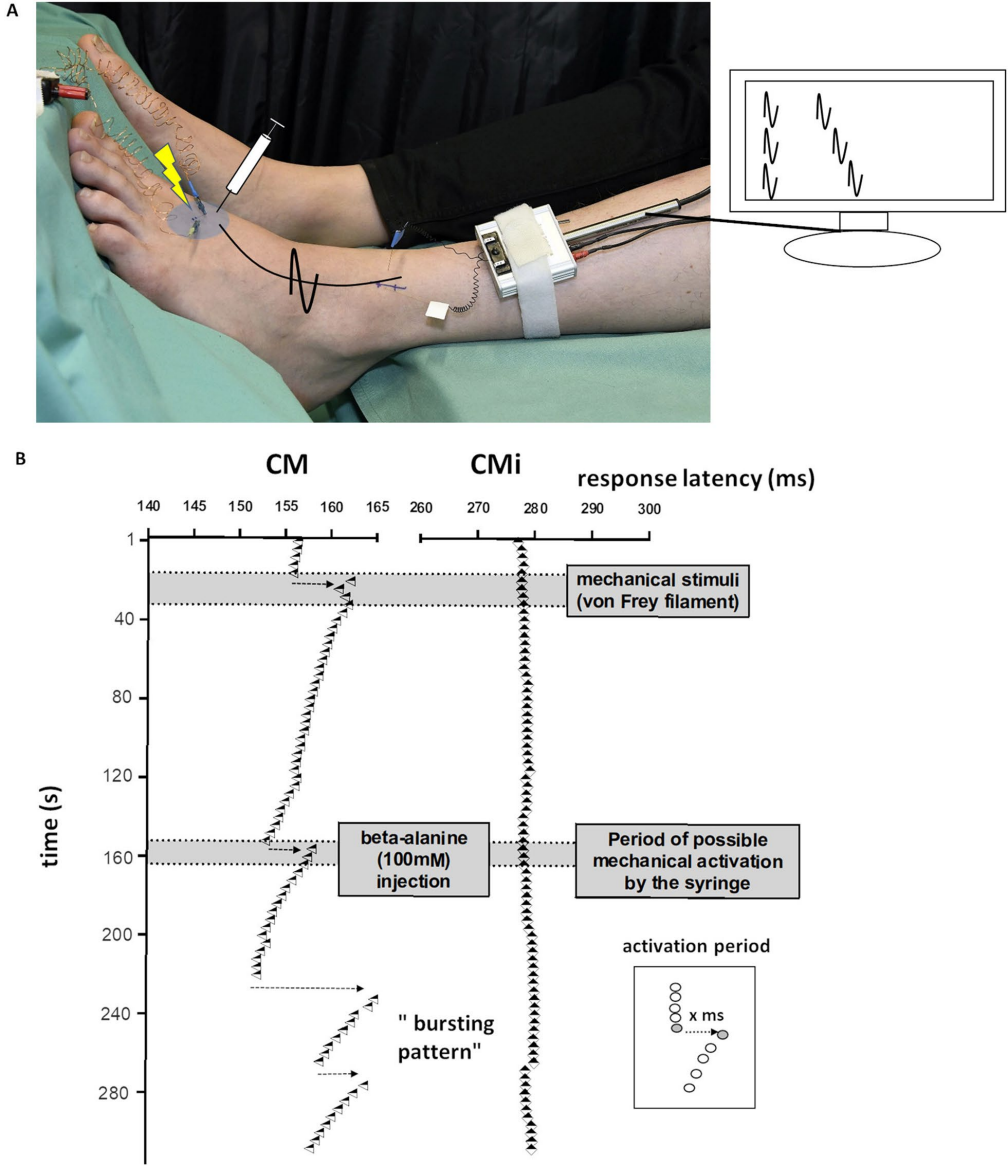
Schematic overview of the microneurography setup and single-fiber responses. **A** A tungsten recording needle (white flag) was inserted into C-fiber fascicles of the superficial peroneal nerve and a reference electrode (blue flag) needle into the skin nearby. A pair of needles was inserted into the previously located innervation territory (gray circle) for intracutaneous stimulation of the recorded C-fibers at a low repetition rate (0.25 Hz). Intracutaneous microinjections of pruritogens were applied in the receptive field of the recorded fibers (gray circle). **B** The latencies of electrically induced action potentials (0.25 Hz) for one CM (triangles) and one CMi (diamonds) fiber are depicted. The CM-fiber shows activation (dotted black arrow) by mechanical (first gray bar) and chemical stimulation in the form of a sudden increase in latency of the electrically induced action potentials, whereas the CMi-fiber does not respond to the mechanical stimuli. Note the regular activation periods in the CM-fiber due to chemical activation with long intervening breaks indicating a “slow bursting pattern”. Only activation periods that occurred in the time frame after the syringe was removed (gray bar) were also counted as such. The cumulative latency shift was assessed as the sum of all individual activation periods of one C-fiber

Microneurography was used to record the action potentials of single C-fibers from cutaneous C-fiber fascicles of the superficial peroneal nerve as previously described [9, 31]. When the inserted tungsten recording needle (Frederick-Haer, Bowdoinham, ME, USA) is placed close to an unmyelinated afferent nerve fiber bundle and has reached a stable position, C-unit innervation territories are detected using a pointed electrode (0.5 mm diameter) delivering electrical pulses. C-fiber units are identified by their low conduction velocity (< 2 m/s). A pair of 0.2 mm diameter needle electrodes (Frederick-Haer) is inserted into the previously located innervation territory (Fig. 1A, gray circle) for intracutaneous stimulation of the recorded C-fibers at a low repetition rate using a Digitimer DS7 constant current stimulator. The signal is amplified, filtered and stored on a computer using custom-written Spike2 software (CED, Cambridge, UK) or DAPSYS and a micro1401 DAC (CED). Single C-fibers were differentiated by their unique conduction latency during continuous low frequency stimulation (0.25 Hz; intensity at least 1.5× individual electrical fiber threshold). After recording C-fiber responses, we used the “marking technique” to characterize the units. This is based on the slowing of conduction velocity when a C-fiber conducts more than one action potential within a short time period, which known as activity-dependent conduction velocity slowing (ADS). The amount of ADS strongly correlates with the number of additional action potentials conducted in the seconds before the electrically induced action potential [9, 32]. This method enables us to determine the chemical responses semi-quantitatively.

To determine the mechanical sensitivity of the recorded C-fibers, we repetitively applied mechanical stimuli using stiff von Frey filaments of 1.2 to 22 g (Stoelting, Chicago, IL, USA) in the receptive fields on the dorsum of the foot in an area of roughly 3 cm around the stimulation needles. Microneurography data were amplified, processed online using DAPSYS and analyzed offline using DAPSYS and Microsoft Excel.

#### C-fiber classification

We assigned C-fibers as mechanosensitive (CM), mechano-insensitive (CMi) and very high threshold (VHT) fibers according to their mechanical responses and electrophysiological properties. C-fibers with an ADS < 5% of their initial latency to an electrical stimulation protocol with rising frequencies (20 pulses at 0.125 Hz, 20 pulses at 0.25 Hz, 30 pulses at 0.5 Hz), a normalization of latency thereafter of more than 24% within 40 s, and a response to < 22 g von Frey stimulation, were classified as CM-fibers. C-fibers with an ADS > 5% and a recovery of < 24% were classified as CMi-fibers or VHT-fibers. We differentiated between the latter by their mechanical response: CMi-fibers show no response to mechanical stimuli whereas VHT-fibers are activated by mechanical stimuli > 10 g von Frey hair filaments [33].

#### Microneurography protocol

After classifying the C-fibers, intracutaneous microinjections of pruritogens were applied to the receptive field of the fibers during electrical stimulation at 0.25 Hz (Fig. 1A, B). After removal of the injection syringe, chemical activation was semi-quantitatively analyzed using the marking technique [34]. We used two parameters based on these latency shifts to quantify activation strength: number of activation periods (summed latency shifts after application of the pruritogens) and cumulative latency (sum of all conduction delays in ms during the chemical responses) [35]. Activation of a C-fiber was assumed if the cumulative latency shift after chemical stimulation exceeded 5 ms. We defined a specific “slow bursting pattern” as at least three repetitions of the following pattern: a sudden latency increase (seen as a shift to the right in Fig. 1B) indicating a preceding train of action potentials within the previous 4 s, followed by at least 20 s of no chemically-induced discharges in five consecutive electrically induced action potentials with normalizing latencies (seen as stepwise latency shifts to the left in Fig. 1B).

### Data analysis and statistics

All data were pre-tested for normal distribution (Shapiro–Wilk test). Normally distributed values are shown as means ± standard errors (SEM) and were analyzed by repeated measures or multi-way analysis of variance (ANOVA) with least significant difference (LSD) post hoc testing. Non-parametric data are shown as medians and quartiles, and were analyzed using the Wilcoxon matched pairs test. For psychophysical data, which were shown in individual values, we used in parallel mean and SEM instead of medians and quartiles for more clarity and to enable easier comparison to other studies in the field, which used means. A P value < 0.05 was regarded as significant. For comparison of numbers of fibers/subjects, we used Pearson’s chi-squared test. All data were analyzed using STATISTICA v7.0 (StatSoft, Tulsa, OK, USA). Graphs and figures were generated with GraphPad Prism v9 (GraphPad Software, San Diego, CA, USA) and Microsoft Excel.

## Results

### Psychophysical study

#### Application mode-dependent itch and pain sensation

To determine whether the magnitude of itch or pain sensations depends on the mode of application, we applied different pruritogens via intracutaneous microinjection or substance-loaded inactivated cowhage spicules. The injection of a substance should lead to homogenous distribution, including deeper skin layers, whereas cowhage spicules allow precise focal and superficial application at higher concentrations [36]. All substances caused itch and pain sensations via both application modes, but in different proportions.

**Fig. 2:**
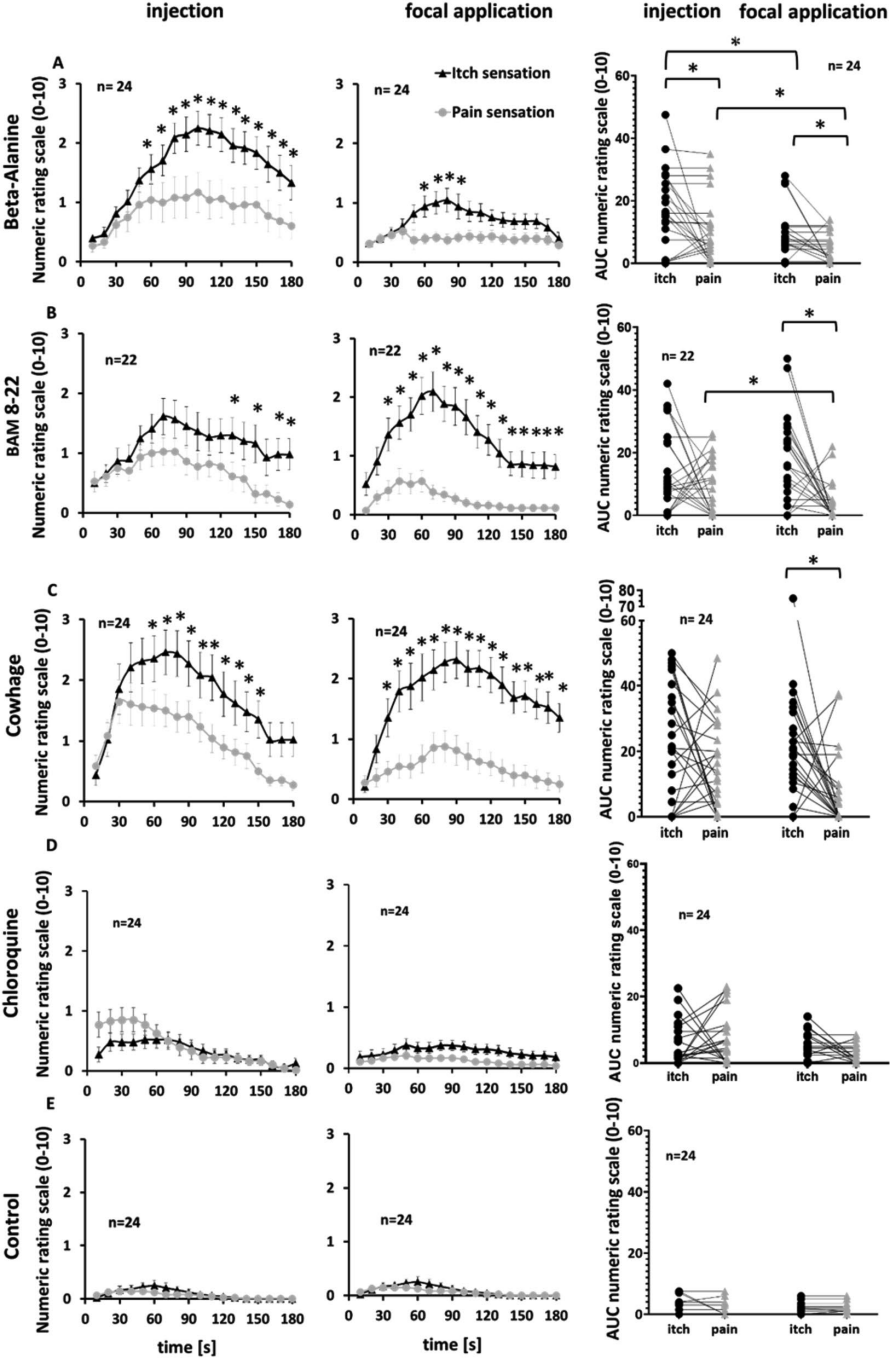
The application route of pruritogens causes different itch/pain rating ratios in human volunteers. The pruritogens were **A** β-alanine, **B** BAM 8-22, **C** cowhage extract and **D** chloroquine and **E** SIF as a control. Itch (black triangles) and pain (gray diamonds) ratings on a NRS from 0 to 10 are depicted for injection (left column) and focal application (right column) during a time series (first and second column) as mean ± SEM and the AUC (third column) for single volunteers. Significant differences are indicated (*P < 0.05)

In the case of β-alanine, most volunteers experienced itch sensations [36] with a rating ≥ 1 following intracutaneous injection (20 of 24 subjects) and focal application (18 of 24 subjects) (Pearson chi-square, p = 0.48). Similarly, pain was reported by 17 of 24 subjects following injection and 11 of 24 following focal application (x^2^_(1)_ = 3.09, p = 0.08). In both application modes, β-alanine evoked more itching than pain over the observation time (AUC injection, itch 18.19 ± 2.45, pain 10.42 ± 2.05; Wilcoxon matched pairs, p = 0.037; AUC focal, itch 8.98 ± 1.61, pain 4.96 ± 0.97; Wilcoxon matched pairs, p = 0.007; Fig. 2A). The maximum itch ratings after injection (NRS 2.6 ± 0.28) were significantly higher than the maximum pain ratings (NRS 1.5 ± 0.23; Wilcoxon matched pairs, p = 0.01). Focal application resulted in lower maximum values but a comparable ratio (NRS itch 1.27 ± 0.21, pain 0.73 ± 0.16; Wilcoxon matched pairs test, p = 0.03; Fig. 2A).

In the case of BAM 8-22, an itch rating ≥ 1 was reported by 18 of 22 subjects, but fewer reported a pain rating ≥ 1, particularly after focal application (injection 14 of 22, focal 8 of 22; x^2^_(1)_ = 3.27, p = 0.07). The injection of BAM 8-22 (AUC itch 14.16 ± 2.75, pain 9.8 ± 2.0; Wilcoxon matched pairs, p = 0.32) caused more intense pain but slightly less itching than focal application (AUC itch 18.25 ± 2.94, pain 3.95 ± 1.33; Wilcoxon matched pairs, p = 0.003; Fig. 2B). Similarly, injections resulted in higher maximum pain scores than focal application (injection itch 2.07 ± 0.3, pain 1.4 ± 0.23; Wilcoxon matched pairs test, p = 0.2; focal itch 2.4 ± 0.33, pain 0.7 ± 0.22; Wilcoxon matched pairs, p < 0.001).

Chloroquine caused substantial pain during injection (mean NRS = 6) but only slightly painful or itchy sensations persisted in a few volunteers (pain ≥ 1 in 7/24, itch ≥ 1 in 10/24; x^2^_(1)_ = 0.82, p = 0.37). Focal application caused pain ≥ 1 in significantly fewer volunteers than injection (focal 2 of 24 subjects vs injection 15 of 24; x^2^_(1)_ = 15.39, p < 0.001). Chloroquine caused in seven subjects slight itch of an NRS rating maximum of 1, which is by our definition the minimal sensation as specified as itch. The maximum itch rating was greater than 1 in only three volunteers (maximum NRS rating of 2 in two volunteers and a maximum NRS rating of 3 in one volunteer). But compared to the other substances are the maximum itch and pain ratings very low (injection itch 0.75 ± 0.16, pain 1.04 ± 0.20; focal application itch 0.48 ± 0.11, pain 0.29 ± 0.07). Given the low itch response to chloroquine, this substance was excluded from further analysis.

The proportional itch and pain sensation was also substantially influenced by the application mode of cowhage. Both methods evoked an itch rating ≥ 1 in the majority of the subjects (injection 18 of 24, focal 21 of 24; x^2^_(1)_ = 1.23, p = 0.27), whereas significantly more subjects reported painful sensations after injection (19 of 24) than after focal application (11 of 24; x^2^_(1)_ = 5.69, p = 0.02). Injection of cowhage extract caused cumulative itch and pain ratings of comparable magnitude (itch 23.25 ± 3.58, pain 15.48 ± 2.78; Wilcoxon matched pairs, p = 0.12). Similarly, the maximum itch ratings (2.9 ± 0.45) and pain ratings (2.13 ± 0.34) were comparable (Wilcoxon matched pairs, p = 0.14; Fig. 2C). In contrast, focal application caused significantly higher cumulative itch ratings (AUC 21.21 ± 3.37) than pain ratings (AUC 7.29 ± 2.33; Wilcoxon matched pairs, p < 0.001; Fig. 2D). Also, the maximum itch rating was significantly higher (2.65 ± 0.34) than the maximum pain rating (0.96 ± 0.25; Wilcoxon matched pairs, p < 0.001).

**Fig. 3:**
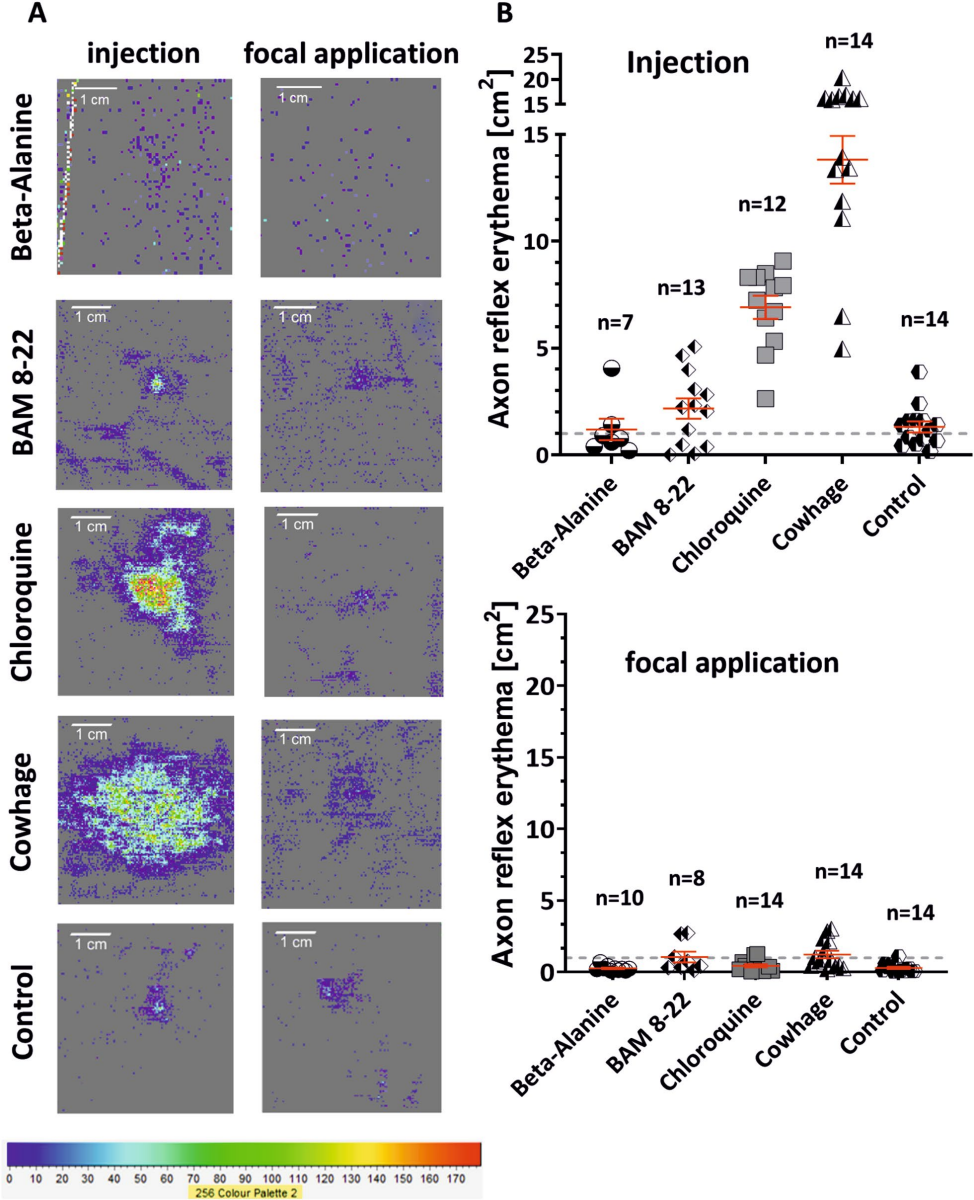
Application modes affect the area of axon-reflex erythema. **A** Laser-speckle imaging after microinjection (left panel) or focal application (right panel) of pruritogens. The intensity of the signal is color coded from light blue to red (color palette at the bottom). **B** Area of axon-reflex erythema after microinjection of the pruritogens (upper panel) and after focal application (bottom panel). A widespread axon-reflex erythema occurs once it reaches a threshold area of 1 cm^2^ (gray dashed line)

During the experimental series comparing the effects of injection versus focal application, the area of axon-reflex erythema was assessed by laser Doppler imaging, which objectively detects the activation of CMi-fibers. The injection of chloroquine and cowhage extract caused widespread axon-reflex erythema, with mean maximum areas around the stimulation site of 6.90 ± 0.55 and 13.81 ± 1.11 cm^2^, respectively. In contrast, focal application caused only local vasodilation, with mean maximum areas of 0.44 ± 0.11 cm^2^ for chloroquine and 1.23 ± 0.26 cm^2^ for cowhage (injection versus focal application, Wilcoxon matched pairs, p < 0.001 for both substances; Fig. 3). Neither β-alanine nor BAM 8-22 induced axon-reflex erythema around the application site, regardless of the application route, indicating a lack of CMi-fiber activation.

#### A-fibers contribute to itch and pain induced by β-alanine

To assess the involvement of C-fibers and/or A-fibers in itch and pain sensations evoked by pruritogens, we applied a selective A-fiber pressure block at the superficial radial nerve. After a mean 49 ± 1 min of pressure application, an A-fiber block with preserved C-fiber conduction was confirmed by the loss of cold sensation but preserved warmth and pinprick sensation.

**Fig. 4:**
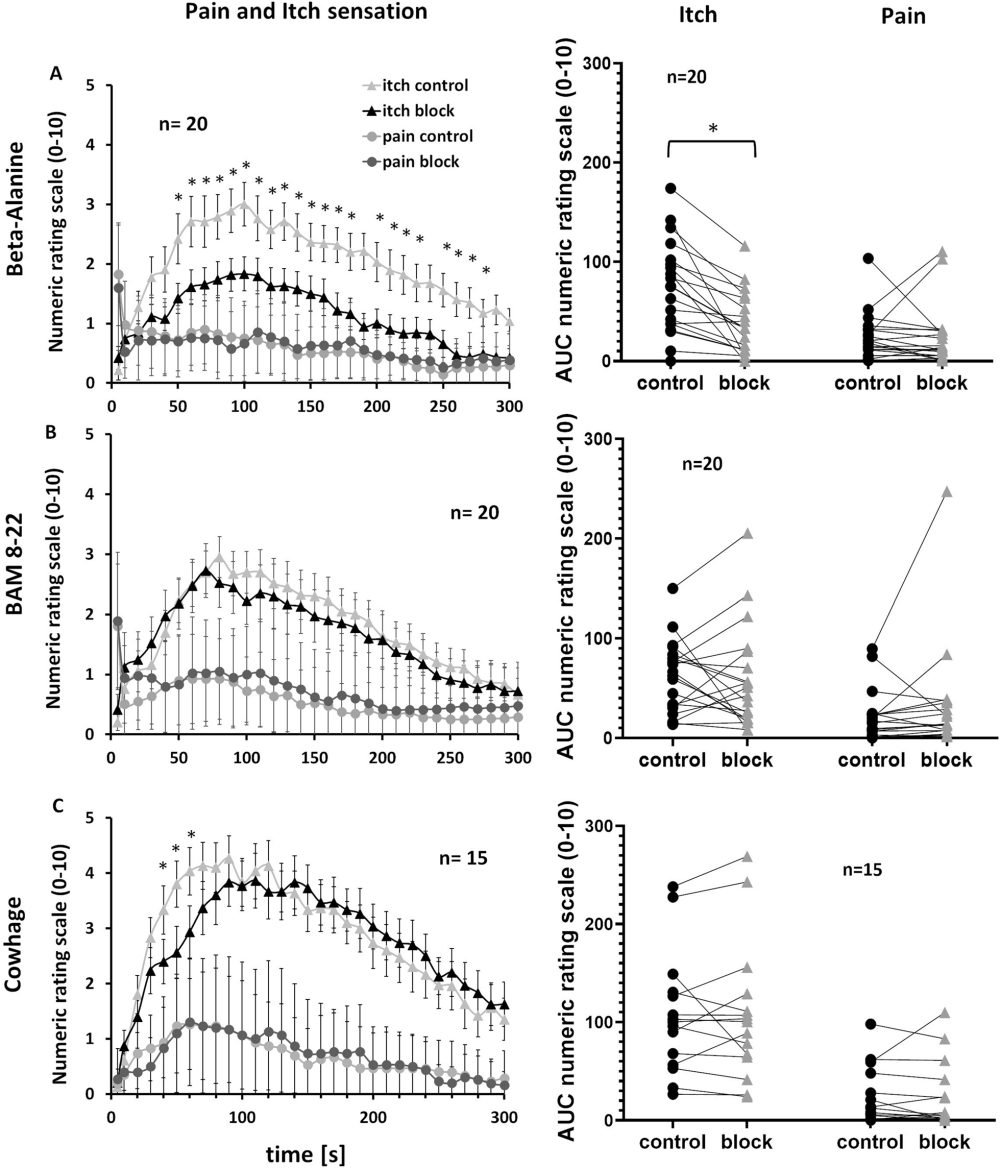
Selective A-fiber nerve block experiments using Beta-Alanine (panels A), BAM 8-22 (panels B) and  Cowhage (panels C) were employed to dissect C- or A-fiber involvement in generation of itch and pain sensation. A selective A-fiber nerve block influences itch sensations only when induced by β-alanine. The first column shows the NRS ratings for itch (triangles) and pain (circles) following pruritogen injection over the course of 300 s (values are means ± SEM) during a selective A-fiber block (dark gray symbols) and control conditions (light gray symbols). The second column shows the AUC of the NRS ratings of all subjects during the A-fiber block (black circles) and without (gray triangles). Significant differences are indicated (*P < 0.05)

The selective A-fiber block reduced itch sensations caused by the injection of β-alanine, but not BAM 8-22 or cowhage extract (Fig. 4). We defined a relevant reduction as a difference of > 5 NRS points in cumulative ratings (AUC). The A-fiber block reduced itch ratings induced by β-alanine in 17 of 20 subjects, whereas pain ratings were lower in only nine of 20 subjects (x^2^_(1)_ = 7.03, p = 0.01). The mean cumulative itch ratings evoked by β-alanine were significantly reduced by the A-fiber pressure block (34.5 ± 7.29 during block vs 70.82 ± 10.90 without; Wilcoxon matched pairs, p < 0.001) indicating that A-fibers are involved in itch sensations induced by β-alanine. The maximum itch rating for β-alanine was also significantly reduced by the A-fiber block (1.83 ± 0.29 during block vs 3.00 ± 0.37 without; Wilcoxon matched pairs, p < 0.001). Pain sensation did not change significantly (AUC 22.57 ± 21.24 during block, 22.96 ± 16.53 without; Wilcoxon matched pairs, p = 0.38; mean maximum pain 1.9 ± 0.3 during block, 2.15 ± 0.24 without; Wilcoxon matched pairs, p = 0.29; Fig. 4A).

The selective A-fiber block also reduced itch sensations caused by the injection of BAM 8-22 in 11 of 20 subjects, but pain was only reduced in three of 20 subjects (x^2^_(1)_ = 7.03, p = 0.01; Fig. 4B). However, there was no effect on the maximum itch sensation (NRS = 3.63 ± 0.23 during block, 3.35 ± 0.31 without; Wilcoxon matched pairs, p = 0.34; Fig. 4B) or the cumulative ratings (54.99 ± 14.79 during block, 58.8 ± 10.82 without; Wilcoxon matched pairs, p = 0.77). Similarly the sensation of pain did not significantly change (NRS = 2.44 ± 0.33 during block, 2.0 ± 0.32 without; Wilcoxon matched pairs, p = 0.15; cumulative pain rating 28.99 ± 37.9 during block, 19.1 ± 17.79 without; Wilcoxon matched pairs, p = 0.08).

The selective A-fiber block had no significant effect on the sensations caused by cowhage extract injection. Changes in itch or pain sensations were only reported by five and seven of the subjects, respectively. There was no significant change in the maximum itch rating (NRS = 4.47 ± 0.47 during block, 4.73 ± 0.4 without; Wilcoxon matched pairs, p = 0.31) or the cumulative rating over time (AUC 76.68 ± 19.94 during block, 76.44 ± 18.51 without; Wilcoxon matched pairs, p = 0.77). Similarly, there was no significant change in the maximum pain rating (NRS = 1.7 ± 0.49 during block, 1.67 ± 0.36 without; Wilcoxon matched pairs, p = 0.83) or the cumulative rating (AUC = 17.54 ± 21.9 during block, 17.34 ± 19.12 without; Wilcoxon matched pairs, p = 0.43; Fig. 4C).

#### TRPA1 and TRPV1 are involved in non-histaminergic itch signaling

**Fig. 5:**
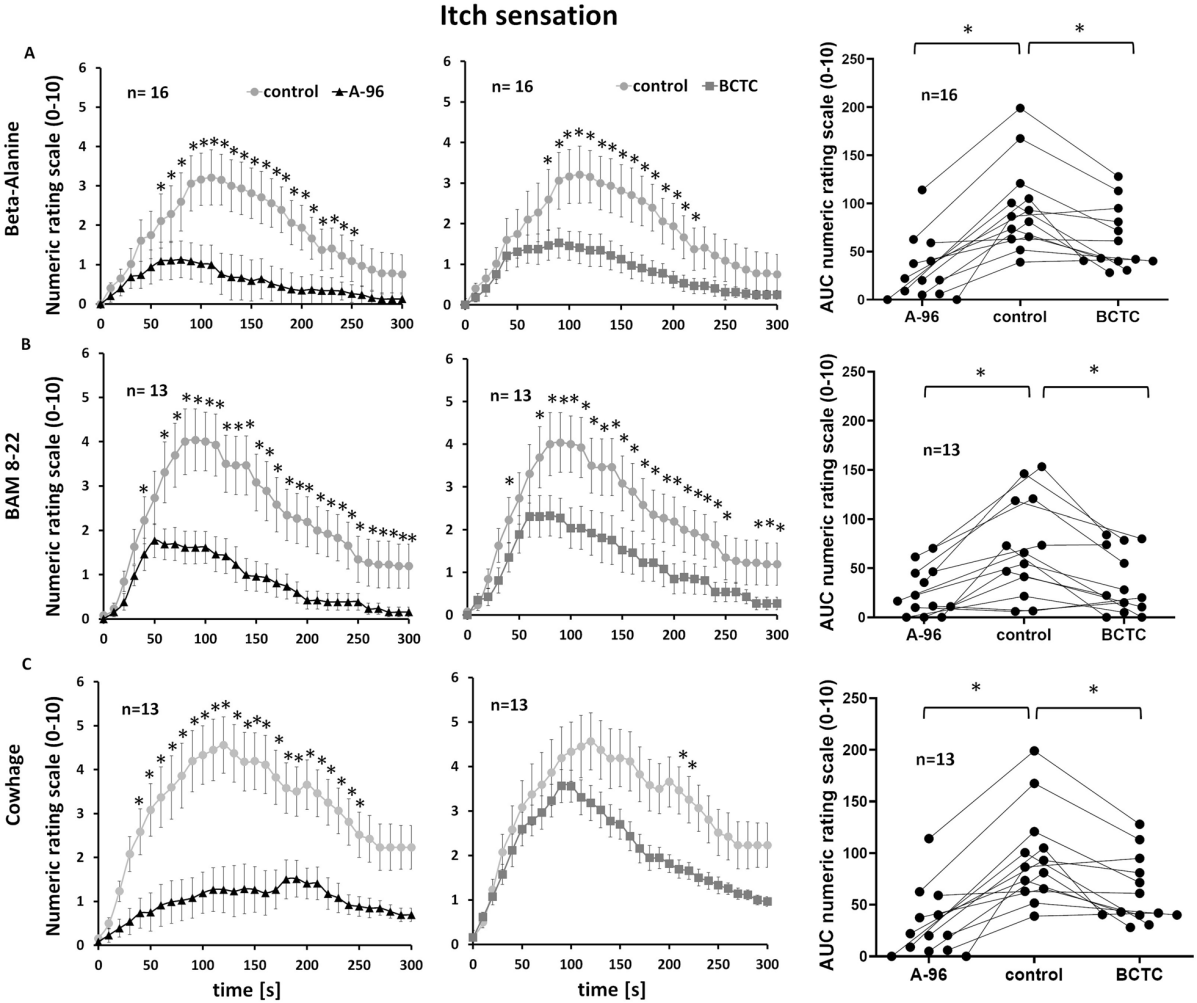
TRPA1/TRPV1 as effector channels for non-histaminergic itch signaling. The involvement of  the ion channels TRPA1 and TRPV1 in itch and pain sensation induced by Beta-Alanine (panels** A**), BAM 8-22 (panels** B**) and  Cowhage (panels** C**) were assesed using respective specific pharmacological blockers A-967079 and BCTC. The first two columns show itch ratings (NRS, values are means ± SEM) for 300 s after the injection of the pruritogens with (black triangles) and without (gray circles) pre-injection of the TRPA1 blocker (A-967079) or the TRPV1 blocker (BCTC). The third shows single values of the AUC for itch ratings provided by each volunteer for control in the middle and with pre-injection of TRPA1 blocker to the left and TRPV1 blocker to the right. Significant differences are indicated (*P < 0.05)

The GPCRs that bind pruritogen ligands are not thought to directly evoke action potentials, but instead trigger second messenger cascades to activate depolarizing ion channels. The primary effector candidates are the *transient receptor potential* (TRP) channels *vanilloid* (V) 1 and *ankyrin* (A) 1. In order to investigate their role in initiating itch signals, we used specific channel blockers: A-967079 for TRPA1 and BCTC for TRPV1. These were pre-injected intracutaneously before intracutaneous injection, at the same skin site, of β-alanine, BAM 8-22 or cowhage extract. For each pruritogen, we compared the number of subjects reporting a less severe itch, defined as a difference > 5 in cumulative ratings between the pre-injection of the channel blocker and the control treatment. Almost all subjects reported a clear reduction in itch sensations induced by β-alanine in the presence of the TRPA1 blocker A-967079 (15 of 16 subjects) and the TRPV1 blocker BCTC (14 of 16 subjects) compared to the control (TRPA1 block, x^2^_(1)_ = 28.23, p < 0.001; TRPV1 block, x^2^_(1)_ = 3.09, p < 0.001). Both channel blockers reduced the cumulative itch sensation caused by β-alanine (BCTC from 56.3 ± 7.4 to 25.08 ± 5.08; Wilcoxon matched pairs, p < 0.001; A-967079 from 56.3 ± 7.4 to 16.84 ± 5.77; Wilcoxon matched pairs, p < 0.001; Fig. 5A). Similarly, the maximum pain ratings were significantly reduced by blocking TRPA1 (from 3.70 ± 1.15 to 1.32 ± 0.27; Wilcoxon matched pairs, p < 0.001) and TRPV1 (from 3.70 ± 1.15 to 1.97 ± 0.95; Wilcoxon matched pairs, p < 0.001).

Both channel blockers also reduced the itch sensation induced by BAM 8-22 (11 of 13 subjects for the TRPA1 blocker; x^2^_(1)_ = 19.07, p < 0.001; 10 of 13 subjects for the TRPV1 blocker; x^2^_(1)_ = 16.25, p < 0.001; zero subjects for the control). The TRPA1 block reduced the cumulative itch rating from 71.34 ± 14.3 to 25.41 ± 6.93 and the mean maximum itch sensation from 4.62 ± 1.4 to 2.12 ± 1.27 (in both cases, Wilcoxon matched pairs, p < 0.001). Similarly, the TRPV block reduced the cumulative itch rating from 71.34 ± 14.3 to 36.33 ± 9.5 and the mean maximum itch sensation from 4.62 ± 1.4 to 2.81 ± 0.4 (in both cases, Wilcoxon matched pairs, p = 0.01; Fig. 5B).

Both channel blockers also reduced the itch sensation induced by cowhage extract (12 of 13 subjects for the TRPA1 blocker; x^2^_(1)_ = 22.29, p < 0.001; 10 of 13 subjects for the TRPV1 blocker; x^2^_(1)_ = 16.25, p < 0.001). The TRPA1 and TRPV1 blocks reduced the cumulative itch rating from 95.84 ± 13.04 to 30.42 ± 9.4 and 62.58 ± 9.44, respectively (Wilcoxon matched pairs, p < 0.001). Interestingly, whereas the TRPA1 blockade reduced the maximum itch rating throughout the evaluation period (from 5.41 ± 0.98 to 2.08 ± 0.43; Wilcoxon matched pairs, p < 0.001), the TRPV1 blockade reduced the maximum itch sensation only after the maximum value was reached (from 5.41 ± 0.98 to 3.88 ± 0.7; Wilcoxon matched pairs, p = 0.01).

#### PGE2 increases pain induced by BAM 8-22 and itch induced by cowhage spicules

**Fig. 6:**
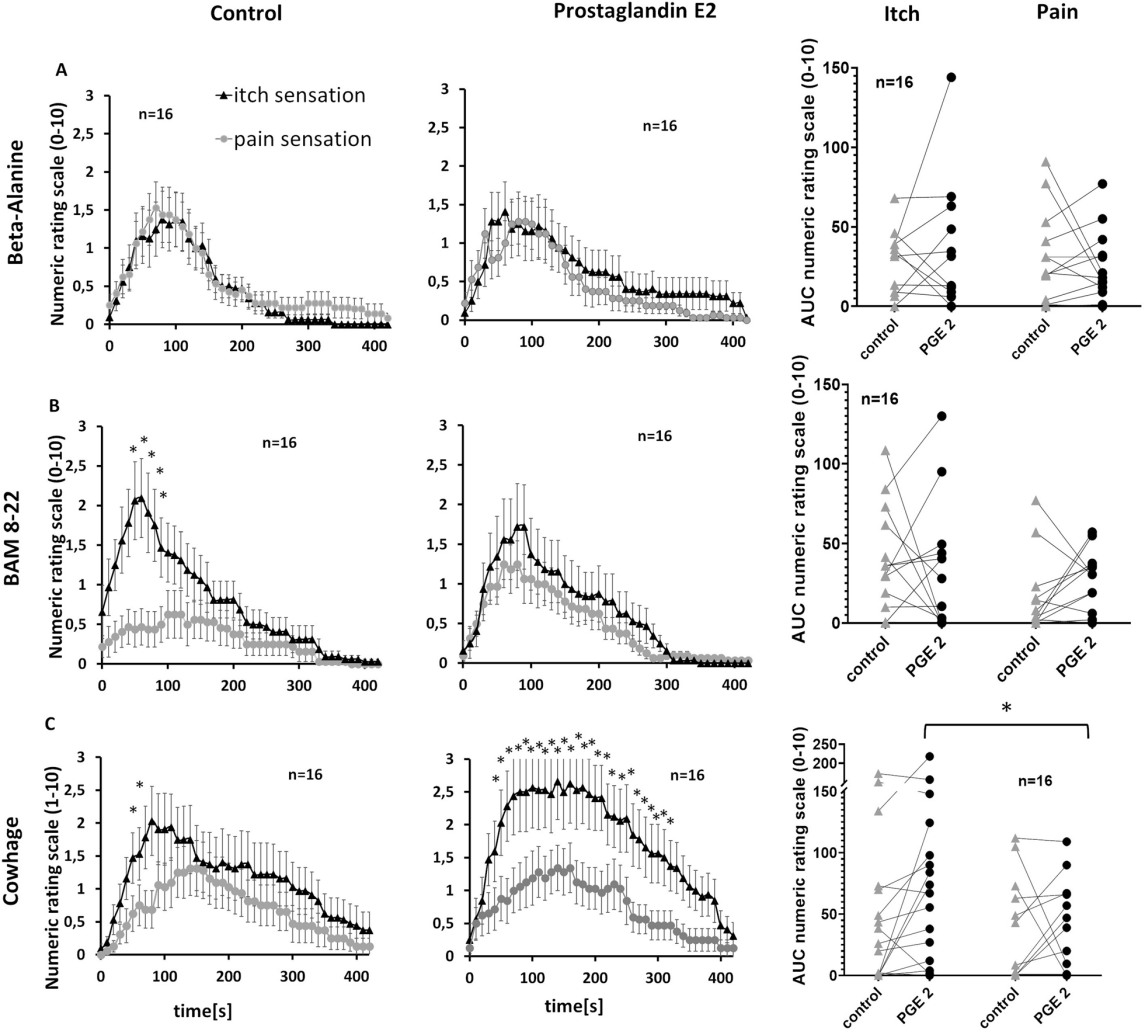
Modulation of non-histaminergic itch sensations by the inflammatory mediator prostaglandin E2 (PGE2). Preinjection of PGE2 was used to asses the effect of PGE2 on perception of Beta-Alanine (panels A), BAM 8-22 (panels B) and  Cowhage (panels C) induced itch and pain. The first two columns show the itch (black triangles) and pain (gray diamonds) ratings (values are means ± SEM) for 420 s after the injection of the pruritogen with the pre-injection of a control solution (left column) or PGE2 (middle column). The third column shows single AUC values for itch and pain ratings provided by single volunteers. Lines connect values form the same volunteer. Significant differences are indicated (*P < 0.05)

We investigated whether inflammatory mediators change the proportion of itch and pain sensations evoked by the injection of β-alanine or BAM 8-22, or the application of cowhage spicules. Accordingly, we injected 100 µL of the control solution, PGE2 (Fig. 6) or bradykinin (Additional file 1: Fig. S1) before the pruritogen at the same skin site.

Neither the control solution nor PGE2 pre-injection changed the itch or pain sensations induced by β-alanine injection, resulting in no significant differences in the number of subjects reporting itch or pain sensations rated ≥ 1 at least once during the observation time (Fig. 6A). Similarly, there was no significant change in the number of subjects reporting itch sensations ≥ 1 caused by BAM 8-22 (eight of 16 in the control group, 10 of 16 pre-injected with PGE2) or in the number of subjects reporting pain ≥ 1 (nine of 16 in the control group, 11 of 16 pre-injected with PGE2). However, PGE2 induced changes in the itch/pain ratio after BAM 8-22 injection. Under control conditions, BAM 8-22 induced significantly more itch than pain sensations in the interval between 50 and 80 s after injection (ANOVA F(42/630) = 2.45, post hoc Bonferroni, p < 0.001; Fig. 6B). The predominance of itch over pain was not observed following the pre-injection of PGE2 (ANOVA F(42/630) = 2.45, post hoc Bonferroni non-significant; Fig. 6B). Similarly, the differences between maximum itch ratings tended to be smaller after the pre-injection of PGE2 (control solution 2.38 ± 0.5, PGE2 1.84 ± 0.52; Wilcoxon matched pairs, p = 0.051). This difference in itch/pain ratio was not caused by changing the magnitude of itch sensations because cumulative itch ratings induced by BAM 8-22 did not change significantly following the pre-injection of control solution (33.09 ± 8.41 to 27.59 ± 9.64; Fig. 6B). However, the pre-injection of PGE2 tended to increase the cumulative pain ratings (control 12.8 ± 5.63, PGE2 20.47 ± 5.05) and maximum pain ratings were slightly higher after PGE2 pre-injection (NRS 1.38 ± 0.28) compared to very low pain ratings after control injection (NRS 1.03 ± 0.30; Wilcoxon matched pairs, p = 0.51).

Itch sensations induced by cowhage were higher at most time points between 50 and 310 s following PGE2 pre-injection (ANOVA F(42/630) = 1.23, post hoc Bonferroni, p < 0.001), resulting in a prolonged maximum itch rating (Fig. 6C). The cumulative itch ratings were higher after PGE2 pre-injection (74.9 ± 15.8) compared to controls (48.75 ± 14.4) whereas the cumulative pain ratings remained in the same range (control = 28.6 ± 10; PGE2 = 31.6 ± 9.2). This indicates a significant difference between itch and pain magnitude after PGE2 pre-injection (Wilcoxon matched pairs, p = 0.01), but not in the control treatment. Similarly, the difference between maximum itch and maximum pain was greater following the pre-injection of PGE2 (itch 3.56 ± 0.51; pain 1.97 ± 0.47; Wilcoxon matched pairs, p = 0.02) compared to the control (itch 2.44 ± 0.55; pain 1.56 ± 0.41; Wilcoxon matched pairs, non-significant). The number of subjects reporting a cowhage-induced itch rating ≥ 1 was higher after PGE2 pre-injection (14 of 16) compared to controls (10 of 16), but the number of subjects rating pain ≥ 1 remained constant (control 10 of 16 compared to PGE2 nine of 16; Fig. 6C).

The results of the psychophysical experiments are summarized in Table 1.

**Table 1:**
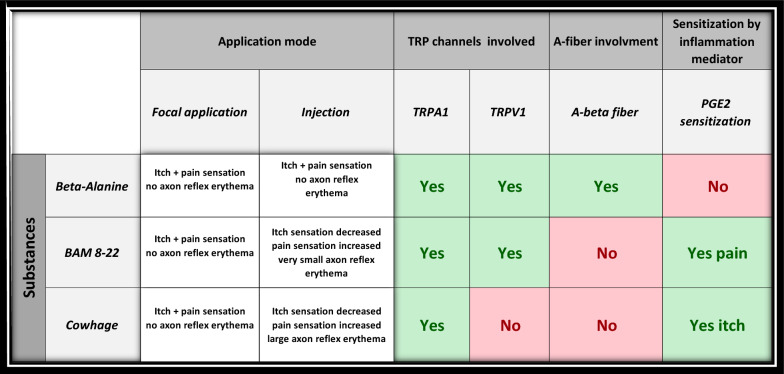
Summary of the psychophysical studies

#### Histamine increases itch sensations in response to painful electrical sine wave stimulation

Electrical sine wave stimulation causes predominantly painful sensations, but some subjects reported burning pain mixed with slight itching. To test whether electrically induced sensations switch from pain to itch following the pre-injection of pruritogens, we assessed the numbers of subjects reporting itch sensations in response to electrical sine wave stimulation before and after pruritogen application. Histamine iontophoresis increased the number of subjects also perceiving itch sensations with a NRS ≥ 1 during electrical sine wave stimulation from four to 12 of 15 subjects (Fisher’s exact test, p < 0.01). Following the injection of BAM 8-22, cowhage extract or β-alanine, electrical sine wave stimulation induced itching in only a few more subjects compared to the absence of pruritogens (Additional file 1: Fig. S2).

### Microneurography

#### Classification of C-fibers

**Fig. 7:**
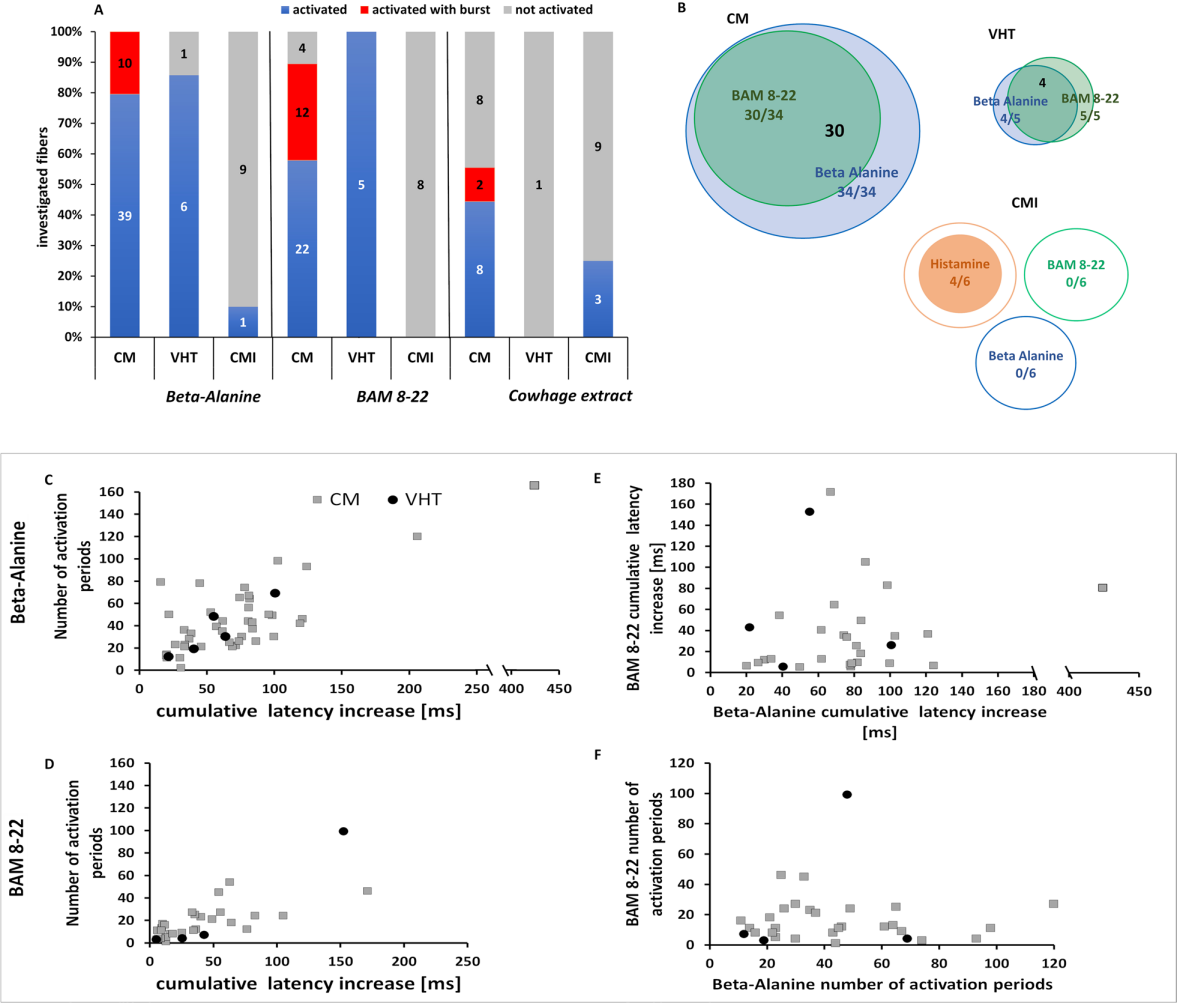
Percentage of C-fibers activated by pruritogens, the number of activation periods and cumulative latency increase. **A** Percentage of C-fibers activated by β-alanine, BAM 8-22 or cowhage extract, showing the percentage of activated (blue), non-activated (gray) and activated with a “slow bursting pattern” (red) mechanosensitive (CM), very high threshold (VHT) and mechano-insensitive (CMi) fibers. The number of fibers is shown within the columns. **B** In a subgroup of the fibers shown in **A**, β-alanine and BAM 8-22 were tested sequentially, and histamine was applied to CMi-fibers. The size of the circle indicates the number of fibers activated by β-alanine (blue), BAM 8-22 (green) or histamine (red) and the numbers are given in the same color. The number of fibers co-activated by β-alanine and BAM 8-22 is shown in bold black. **C**–**F** Number of activation periods (y-axis) and cumulative latency increase (x-axis) during activation after treatment with either **C** β-alanine or **D** BAM 8-22. **E** Cumulative latency increases during activation caused by BAM 8-22 and cumulative latency increase during activation by β-alanine only in those fibers in which activation by both substances could be quantified. **F** Activation periods triggered by BAM 8-22 and β-alanine in those fibers in which activation by both substances could be quantified. In **C** and **D**, more fibers are depicted than in **E** and **F** because the responses could not be reliably quantified or both injections performed in all fibers

We used single nerve fibre recordings in healthy volunteers (microneurography) to investigate which C-fibre subtypes were activated by the intracutaneous microinjection of non-histaminergic pruritogens into their receptive field. We recorded 70 units from 20 healthy young subjects (four males and 16 females, mean age 23 years) over conduction distances of 53–180 mm, revealing 50 CM-fibres, 14 CMi-fibres and seven VHT fibres. The intracutaneous microinjection of β-alanine activated all 49 CM-fibres, six of the seven VHT fibres (86%), but only one of the 10 CMi-fibres (10%) we tested. Similarly, BAM 8-22 activated 34 of the 38 CM-fibres (89,5%), all five of the tested VHT fibres and none of the eight CMi-fibres (Fig. 7A). We also found that 30 of 34 CM-fibres (88,2%) and four of five VHT-fibres (80%) were activated by the injection of either β-alanine or BAM 8-22, whereas none of six tested CMi-fibres responded to either pruritogen. Four of these six CMi-fibres responded to histamine (Fig. 7B).

#### Cowhage extract activates polymodal C-fibres and sleeping C-fibres

Previous work has shown that cowhage spicules activate CM- but not CMi-fibres [4]. We therefore tested, for the first time in humans, whether the intracutaneous injection of cowhage extract has the same effect. We found that the injected extract activated 10 of 18 tested CM-fibres (56%) and three of 12 tested CMi-fibres (25%). Only one VHT-fibre was examined and this did not respond to the cowhage extract.

#### CM-fibers are activated more potently by β-alanine than BAM 8-22

To determine any differences in the magnitude of chemical responses evoked by β-alanine and BAM 8-22, we assessed the discharge magnitudes of single nerve fibers in a semi-quantitative manner. The direct analysis of discharges via automatic spike sorting is often challenging in human microneurography due to the simultaneous recording of several nerve fibers (including sympathetic efferent fibers) and a low signal-to-noise ratio. Therefore, we compared the cumulative latency shifts and activation periods as indirect markers of activation strength after chemical stimulation. One activation period is a sudden shift in the electrically induced action potential to a greater latency, the magnitude of which correlates roughly to the number of action potentials discharged in the preceding 4 s (inset Fig. 1). These latency shifts were then summed to determine the cumulative latency. Both the activation period and cumulative latency provide a semi-quantitative activation magnitude for human sensory C-fibers.

CM-fibers responded to β-alanine with 42.9 ± 4 activation periods and a cumulative latency shift of 75.5 ± 10.25 ms. BAM 8-22 was less potent, triggering 16.89 ± 2.23 activation periods and a cumulative latency shift of 35.16 ± 6.2 ms (Wilcoxon matched pairs, p < 0.001 for both parameters). We were able to record from only five VHT-fibers, which showed no differences in the response to β-alanine (n = 5) or BAM 8-22 (n = 4). We observed 35.6 ± 10.32 activation periods in response to β-alanine, and 28.25 ± 23.60 in response to BAM 8-22 (Mann–Whitney U-test, p = 1.0). The cumulative latency shifts were 56.54 ± 13.18 ms for β-alanine and 56.62 ± 32.9 ms for BAM 8-22 (Mann–Whitney U-test, p = 1.1). We observed no differences in the cumulative latency shifts and number of activation periods when comparing VHT-fibers and CM-fibers. Only one of the 10 tested CMi-fibers was clearly activated by β-alanine (66 activation periods; cumulative latency shift 102 ms) whereas BAM 8-22 did not activate any CMi-fibers (n = 8) (Fig. 7C, D).

In a smaller population of C-fibers, we serially injected both β-alanine and BAM 8-22 at two different skin sites within the receptive field. In CM-fibers (n = 34), the injection of β-alanine triggered a stronger response (45 ± 5.29 activation periods, cumulative latency 90.36 ± 14.67 ms) than BAM 8-22 injection (15.81 ± 2.2 activation periods; cumulative latency 36.56 ± 7.3 ms) (Wilcoxon matched pairs, p < 0.001 for both parameters). We observed no clear clusters of CM-fibers that responded potently to either β-alanine or BAM 8-22 alone (Fig. 7E, F). The few recorded VHT-fibers did not show a clear preference for either pruritogen.

#### Distinct discharge patterns in human C-fibers evoked by β-alanine, BAM 8-22 and cowhage extract

**Fig. 8:**
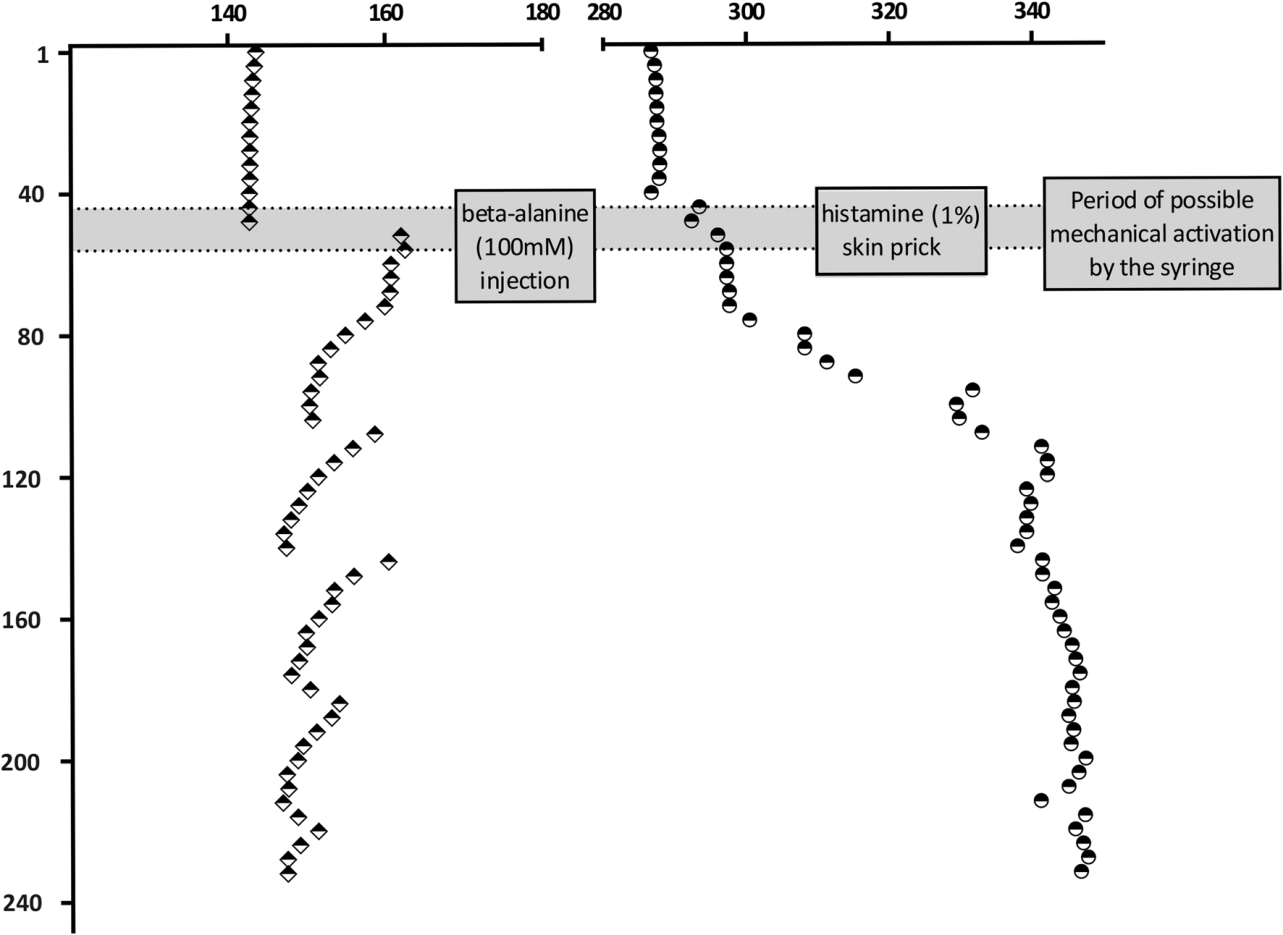
The slow bursting pattern versus continuous activation. The CM-fiber on the left has a stable latency of 144 ms whereas the CMi-fiber on the right has a slower stable latency of 288 ms. The C-fibers were recorded at two different sites. The gray bar shows the time from injection (first dashed line) to the time of removal of the needle (second dashed line). We injected β-alanine for the CM-fiber and introduced a histamine skin prick into the receptive field of the CMi-fiber. During this time, mechanical activation cannot be distinguished from chemical activation. The “slow bursting pattern” can be observed in the CM-fiber response to β-alanine, differing from the irregular response of the CMi-fiber to histamine

We observed a distinct pattern of latency shifts in a subpopulation of CM-fibers after the injection of β-alanine or BAM 8-22, indicating a distinct discharge pattern was evoked by these pruritogens. During microneurography experiments, it is often impossible to asses discharge patterns directly due to extracellular multi-fiber recordings with a low signal-to-noise ratio, which cause spike sorting algorithms to fail. However, the magnitude of sudden latency increases (markings) correlates with the number of previously discharged action potentials [33]. We therefore observed a pattern consisting of substantial latency shifts to the right followed by normalization of the latency, indicating that trains of action potentials were discharged within 4 s followed by no activity for the time of latency normalization. This “slow bursting pattern” consists of activity over approximately 4 s and silent phases each lasting at least 20 s repeated at least three times (Fig. 8).

The injection of β-alanine into the receptive field caused this slow bursting pattern in 10 of 34 activated CM-fibers (20.4%). Two of 10 activated CM-fibers (20%) responded to cowhage extract injections in the same manner. However, this increased to 12 of 34 activated CM-fibers (35.3%) in response to BAM 8-22. A similar bursting pattern was not observed in any of the VHT or CMi-fibers (Fig. 7).

## Discussion

We have shown that non-histaminergic itch signaling in humans cannot be explained solely by the labeled line hypothesis, but instead is based on conceptually different signaling mechanisms relating to the labeled line, spatial contrast, population coding and discharge pattern coding hypotheses, depending on the nature of the pruritogen.

### Population coding is involved in itch signaling induced by β-alanine

Our microneurography experiments showed that all tested CM-fibers were activated by β-alanine and most were strongly activated relative to the definition of “significant activation” in our previous study using cowhage spicules [4]. Assuming that β-alanine activates human nerve fibers exclusively via MrgprD, it is unlikely that only a small subset of those peripheral C-fibers expresses this receptor. Furthermore, we did not observe a subgroup of CM-fibers especially responsive to β-alanine as it was found in non-human primate [10, 19]. The labeled line hypothesis is thus unlikely to explain itch signaling induced by β-alanine specifically via MrgprD or in general.

Recent RNA sequencing (RNA-Seq) studies revealed differences between sensory DRG neurons in humans and mice. In contrast to our findings in humans, MrgprD in mice is expressed by a very small subgroup of DRG neurons [37] whereas most nociceptive human DRG neurons express TRPV1 and about 36% of them express MrgprD [19]. The presence of MrgprD on more than one third of human nociceptive neurons together with the strong activation of CM-fibers by β-alanine argues against both spatial contrast by sparse receptor expression and a labeled line of MrgprD expression on a small subset of fibers as major mechanisms underlying the itch signaling induced by β-alanine. CMi-fibers were not activated by β-alanine injections, and accordingly we and others observed no widespread axon-reflex erythema, which in humans is dependent on CMi activation [3, 15, 19].

In our psychophysical experiments, β-alanine induced more intense itch than pain sensations regardless of the application mode as it was described in previous publication [15, 38]. Injection floods the tissue and activates more nerve fibers than focal application using inactivated cowhage spicules. According to the spatial contrast theory, injection should trigger a greater pain component within the mixed itch/pain sensation than focal application. The same itch/pain ratio with injection and focal application thus argues against spatial contrast as the major signaling mechanism for itch induced by β-alanine. The spatial contrast theory would also explain a larger pain component in the mixed sensation after nociceptor sensitization, as previously observed for the pre-injection of bradykinin before histamine application [39]. However, the pre-injection of PGE2 or bradykinin (Additional file 1) as a sensitizing agent did not push the mixed itch/pain sensation towards more pain. A selective pressure nerve fiber block showed that A-fibers are involved in itch sensations evoked by β-alanine. Accordingly, assuming that β-alanine activates human C-afferent fibers via MrgprD, we suggest that some subpopulations of human sensory A-fibers also express MrgprD.

**Fig. 9:**
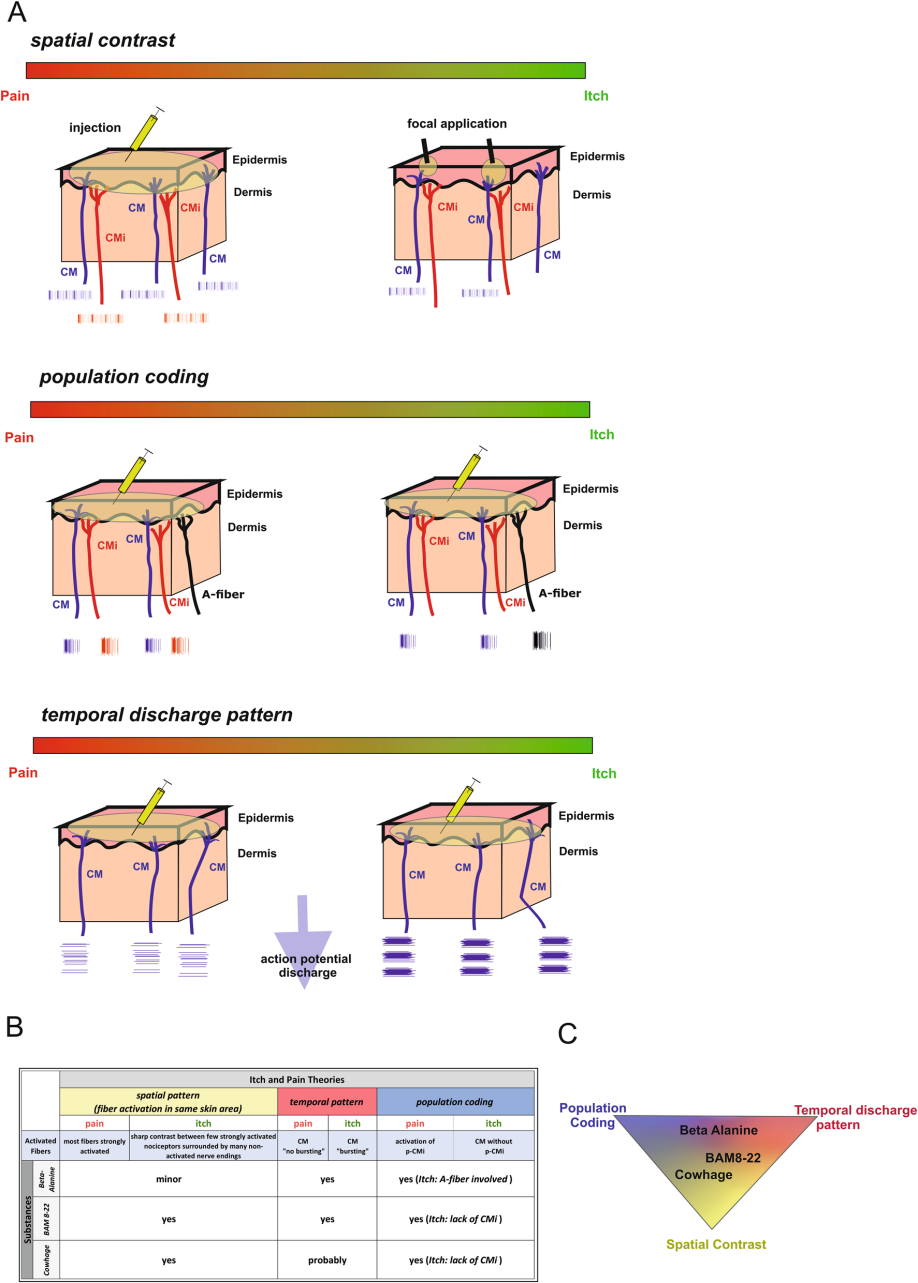
Shows a mechanistic illustration of different itch theories related to our results. **A** From top to bottom is shown: spatial contrast theory, population coding theory and temporal discharge pattern theory. For each theory, a skin section (Epidermis, Dermis) is shown for itch and pain and illustrated by the bar above with the overlapping gradient from pain (red) to itch (green). The activation pattern of the free nerve endings in the skin (CM-fibers: blue; CMi-fibers: red and A-fibers: black) is shown in the colour of the respective fiber typ. (1) Spatial contrast theory: activation of major C-fibers leads to pain; highly activated C-fibers in contrast to silent C-fibers leads to itch (injection vs. focal). (2) Population coding: The activation of CM- and CMi-fibers leads to pain; the activation of A-fibers and/or the lack of CMi-fibers activation leads to itch. (3) Temporal discharge pattern: no bursting discharge in CM-fibers leads to pain and bursting discharge in CM-fibers leads to itch. **B** Combinations of different itch and pain signaling theories potentially explaining the effect of the non-histaminergic pruritogens β-alanine, BAM 8-22 and cowhage. **C** The proximity of substances to the different theories (in the corners) shows, based on our results, the applicability of that theory.

Our results thus far indicate that β-alanine strongly activates the majority of CM-fibers and also some A-fibers but no CMi-fibers, challenging the current status of CMi-fibers as the major human chemo-nociceptors [34]. Previous studies involving chemical CMi activation in humans clearly show an association with pain but not itch sensations, with the exception of histamine-responsive CMi-fibers [18, 34, 35, 40]. CMi-fibers are not homogeneous and can be subdivided into histamine-responsive fibers with huge receptive fields (His-CMi) and highly capsaicin-responsive CMi-fibers [34]. The activation of His-CMi-fibers evoke a greater itch component whereas histamine unresponsive fibers (p-CMi) evoke a greater pain component [34]. The chemical activation of p-CMi-fibers therefore appears to induce pain regardless of concomitant CM- or A-fiber activation. Ligands that activate more CM-fibers and fewer p-CMi-fibers evoke mixed sensations of pain and itching. Accordingly, the chemical activation of CM-fibers possibly together with A-fibers but without p-CMi-fibers may represent an aspect of itch signaling via population coding, which is perceived as an itch sensation. Itch perception is suppressed and replaced by pain when p-CMi-fibers are also chemically activated, or if many CM- and A-fibers are activated by other modalities, such as mechanical stimuli induced by scratching. We therefore propose that itch sensations triggered by β-alanine are signaled by population coding in the form of CM- and A-fiber activation without a CMi-fiber component, together with discharge pattern coding as described below (Fig. 9).

### Spatial contrast is involved in itch signaling induced by BAM 8-22

In contrast to β-alanine, the focal application of BAM 8-22 caused more itching and less pain compared to intracutaneous injection. This may reflect the spatial contrast between strongly-activated fibers and non-activated fibers innervating the same site, causing a mismatched signal that is interpreted centrally as an itch [41]. Also previous studies of other groups showed that application of BAM 8-22 via spicules caused more itch then pain [42, 43]. Another less focal application via 25 pricks evoked more pain and less itch [43]. The fact that β-alanine caused more pain following the pre-injection of PGE2 also supports a spatial contrast signaling mechanism for BAM 8-22. PGE2 presumably sensitizes C-fibers, especially CMi-fibers, and thus additional nociceptors could be activated by β-alanine, resulting in the elimination of the mismatched signal and more pain.

Our microneurography experiments showed that BAM 8-22 injection activated nearly all CM-fibers but no CMi-fibers. Assuming that fiber activation is dependent on MrgprX1, the human homologue of MrgprA1 [44], it is likely that most CM-fibers display this receptor. CM-fibers are also activated by noxious mechanical stimuli and often by noxious heat. Their discharges are responsible for setting the heat pain threshold during psychophysical thermal testing [45]. Human neurons expressing MrgprX1 therefore do not form a special subgroup or a labeled line for itch signaling. However, fewer CM-fibers are activated strongly by BAM 8-22 than by β-alanine, which might contribute to a spatial contrast effect even when BAM 8-22 is injected, thus causing some itch sensation.

Selective A-fiber pressure block experiments suggested that A-fiber activation is not necessary for the BAM 8-22 induced itch sensation. However, some CMi-fibers were activated spuriously by BAM 8-22, either directly or due to histamine released from mast cells in response to BAM 8-22 [46, 47]. Accordingly, we observed a small axon-reflex erythema around the BAM 8-22 application site. However, these effects were much lower than the response to capsaicin and histamine, and CMi-fiber activation is probably negligible in terms of producing a conscious sensation. Accordingly, spatial contrast may combine with population coding to signal itch sensations induced by BAM 8-22, reflecting the lack of p-CMi input as discussed above for β-alanine. We propose that itch signaling induced by BAM 8-22 involves spatial contrast supported by population coding (absence of p-CMi input) together with discharge pattern coding as described below (Fig. 9).

### The same CM-fibers are activated by β-alanine and BAM 8-22

The stimulation of CM-fibers with either β-alanine or BAM 8-22 resulted in different magnitudes of activation, ranging from spurious responses that are unlikely to result in any conscious sensation to strong activation lasting several minutes, which is likely to be perceived as itching or pain. The time course of CM-fiber activation roughly correlated with the duration of itch sensations in the psychophysical experiments. Generally, the activation magnitude (especially the number of activation periods) was lower for BAM 8-22 than β-alanine, but we could not identify any fiber clusters responding preferentially to either pruritogen. This contradicts the results of single nerve fiber recordings in nonhuman primates [10] but agrees with in situ hybridization data on human DRG neurons, where ~ 90% of the MrgprD^+^ neurons express MrgprX1 and vice versa [19]. In nonhuman primates, mechano-sensitive C-fibers can be classified by their response to a stepped heat stimulus as quick (QC) and slow (SC) C-fibers [10]. BAM 8-22 preferentially activates SC-fibers whereas QC-fibers respond better to β-alanine [10]. We were unable to distinguish SC and QC-fibers due to the technical limitations of microneurography, but given that no CM-fibers responded exclusively to each pruritogen it is unlikely that human QC and SC populations have the same chemical responsiveness as in nonhuman primates. It is possible that VHT-fibers (first described in pigs) resemble monkey SC-fibers because other properties are similar, such as ADS. Human VHT-fibers have similar properties to their porcine counterparts (our unpublished results) but there are comparatively few of them. We found that the response of human VHT- and CM-fibers to BAM 8-22 and β-alanine was indistinguishable, suggesting functional differences between fiber subclasses when comparing humans and nonhuman primates. Notably, nonhuman primates have hairy skin, differing in structure from human skin, whereas the skin of domestic pigs is much more similar to human skin (with a thick fatty layer below the dermis compensating for the absence of fur). This may contribute to the specialization of heat-responsive nociceptors in different species.

### Spatial contrast or population coding: cowhage extract injection activates sleeping nociceptors

Numerous studies have shown that the application of cowhage spicules causes itch sensations with some additional components of pricking or burning [22, 48, 49]. Our previous microneurography study demonstrated that all tested CM-fibers, but not CMi-fibers, are strongly activated by cowhage spicules [4]. No widespread axon-reflex vasodilation was observed [50]. In contrast, here we demonstrated that the injection of cowhage extract leads to a widespread axon-reflex erythema, a higher proportion of pain in the overall sensation, and the activation of single CMi-fibers.

The spatial contrast theory explains the switch to less itching and more pain when cowhage extract is injected. However, population coding might also play a role, as discussed above. The injection of cowhage extract reached and activated some CMi-fibers in deeper skin layers, as shown by microneurography and indicated by the widespread axon-reflex erythema. Injection also increased the pain component of the mixed sensation. Similarly, in one of our previous studies, LPA activated CMi-fibers when injected but not when applied via cowhage spicules [18], indicating that spicules do not achieve a sufficient concentration of the pruritogen deep enough into the skin to activate CMi-fibers [18]. This may explain our finding that PGE2 did not increase the pain component induced by cowhage spicules. Although CMi-fibers are efficiently sensitized by PGE2 injection, mucunain does not reach them when applied via spicules. The recruitment of potentially PGE2-sensitized CM-fibers may facilitate population coding as discussed above, leading to the observed increase in the itch sensation following PGE2 pre-injection.

Our microneurography experiments described herein yielded fewer activated CM-fibers compared to our previous study, in which cowhage spicules activated all CM-fibers [4]. This may reflect the different axonal tree morphologies of CM- and CMi-fibers. Injection favors the simultaneous activation of all axonal branches within the small receptive field of a CM-fiber, presumably followed by the rapid influx of calcium resulting in desensitization, as seen also with capsaicin injections [51, 52]. The huge receptive fields of CMi-fibers are only partly covered by the small injection bleb, possibly resulting in successive activation of different branches during diffusion and long-lasting activation, as similarly observed with capsaicin injection [52, 53]. In contrast to injection, focal application leads to a very high focal concentration, which may be sufficient to activate the nearest nerve fibers but not those further away (particularly if the substance diffuses very slowly or a high concentration is needed for activation). Accordingly, microneurography with repeated application of cowhage spicules sometimes resulted in the strong activation of individual CM-fibers whereas others were not activated at all [4].

We also used a selective A-fiber pressure block to demonstrate the negligible involvement of A-fibers in cowhage-induced itch (based on mean NRS values, and individual subjects). Using a similar A-fiber blockade, others have demonstrated that A-fibers contribute to the itchy or burning and pricking sensations induced by cowhage spicules [5]. High variability between individuals might contribute to these discrepancies but the three studies together show that more volunteers show no effect following the A-fiber block than those with a reduced itch response to chemical stimulation during the block. Further experiments using microneurography of A-fibers would be highly interesting to prove activation of A-fibers in non-histminergic itch especially in beta alanine and cowhage.

### Intracutaneous chloroquine induce spurious itching in few healthy human subjects

In mice, chloroquine injection causes strong itch sensations by activating MrgprA3 [14]. In contrast, the injection of chloroquine in our human volunteers caused strong pain during injection, which subsided within seconds, but only caused slight itch in three of 24 volunteers and in eight spurious itch rating of 1. Focal application of chloroquine produced hardy any sensations. Recently activation of TRPA1 by chloroquine has been described in vitro. This might explain the injection pain with subsequent desensitization of the nerve fibers by chloroquine [54]. In black Africans, anti-malaria medication containing chloroquine was found to induce itching [14, 55], but in those cases chloroquine was applied systemically. Thus it might be that differences in skin between black Africans and Caucasian people account partly for the different sensation of itch, but other mechanisms might play a role, too. If chloroquine is injected, the skin is flooded with a substance activating TRPA1 which could lead to overload of the nerve fibers with calcium and subsequent desensitization. This would explain the significant injection pain without following major sensations of itch.

### A potential role for discharge pattern coding in itch sensations

Our microneurography recordings showed that a slow bursting pattern was evoked by β-alanine, BAM 8-22 and cowhage extract in 24%, 35% and 20% of analyzable CM-fibers, respectively. This pattern consists of discharges within ~ 4 s followed by a break of 20–90 s repeated at least three times. The pattern may create a spatial contrast effect over time by asynchronous bursting in several fibers. The proportion of bursting fibers was highest among those activated by BAM 8-22, and this effect may therefore significantly contribute to itch signaling induced by BAM 8-22.

A bursting pattern induced by cowhage spicules was previously described in monkey CMH-fibers [22]. However, the discharge pattern featured shorter discharge breaks of ~ 10 s [22]. We use the marking method to assess activity during microneurography experiments. This means we use latency changes in electrical test pulses of 0.25 Hz to detect previous fiber activation. Our observation window is therefore fixed to 4 s, which hinders the observation of bursts with short intervals. Interestingly, in monkey single nerve fiber recordings, a CMH unit that responded to cowhage with a bursting pattern responded to a heat stimulus with a non-bursting pattern featuring higher discharge frequencies [22]. Different sensation qualities may therefore be coded with specific discharge patterns by the same nerve fibers. Indeed, the same neuron population can evoke itch or pain behavior in mice when activated via metabotropic or ionotropic receptors, respectively[23]. Temporal aspects of neuronal discharges influence the transmission of potential itch signals in the spinal cord. For example, the spinal itch relay seems to require a higher frequency in a burst-like pattern of GRP^+^ neurons, which activate the tertiary GRP receptor neurons [24].

### TRPA1 and TRPV1 are involved in itch signaling induced by BAM 8-22, β-alanine and cowhage in humans

The role of TRP channels in itch signaling has been explored mainly in murine models [56]. In humans, we found that itch sensations triggered by BAM 8-22 and β-alanine were influenced strongly by the pharmacological blockade of receptors TRPA1 and TRPV1, whereas cowhage-induced itch was mainly influenced by the blockade of TRPA1. RNA-Seq data reveal major differences in TRPV1/TRPA1 expression between humans and mice, so we will focus our discussion on the human receptors. Human TRPV1 is expressed on the vast majority of nociceptors and in situ hybridization showed that nearly all human DRG neurons expressing MrgprX1 and MrgprD also express TRPV1 [19]. RNA-Seq data revealed a substantial overlap between TRPV1 and TRPA1 expression in human DRGs. Furthermore, the topical application of capsaicin as a desensitizing agent reduced non-histaminergic itch evoked by β-alanine, BAM-8-22 and cowhage [57, 58]. The therapeutic blockade of TRPV1 or TRPA1 may therefore offer opportunities for the treatment of chronic non-histaminergic itch.

### Electrical sensitization in humans is not caused by β-alanine, BAM 8-22 or cowhage

Electrical stimulation using sinusoidal pulses selectively activates C-fibers at a certain intensity and causes burning pain [59]. In patients with atopic dermatitis, this type of electrical stimulation causes itching in addition to pain [60]. We therefore investigated whether non-histaminergic pruritogens or histamine can cause a similar switch from electrically induced pain to a mixed pain/itch sensation. Interestingly, we found that only the application of histamine caused a switch from pure pain to a mixed itch/pain sensation.

A specific subgroup of CMi-fibers shows a long-term and vigorous response to the iontophoresis of histamine [9] and generates spinal sensitization [61]. Spinal sensitization causing an itch sensation in response to a non-itch inducing stimulus is therefore likely to be initiated by the activation of those histamine-responsive CMi-fibers. Given that neither β-alanine nor BAM 8-22 caused such a switch in sensation, we assume that the spinal and central pathways of non-histaminergic itch differ from those activated by histamine.

## Conclusion

Human itch signaling is a complex process that cannot be solely explained by a labeled line. The differentiation between itching and pain seems to require a combination of mechanisms, including spatial contrast, population coding and potentially also specific discharge patterns. These findings influence the selection of treatment targets for chronic pruritus. Regarding clinical implications, it might be concluded that blocking specific receptors might not be beneficial for many chronic itch conditions, since any activation including mechanical activation of a broad range of different nociceptors might lead to itch. Thus, it might be helpful to include the idea of changing excitability and discharge properties or patterns of peripheral nerve fibers in the search for anti-pruritic medications Our results also influence the terminology of human sensory C-fibers, particularly the clear distinction between nociceptors and pruriceptors, because the same fiber type can signal both itch and pain (e.g., chemical itch and heat pain). During chemical activation, CM-fibers seem to signal itch sensations whereas p-CMi-fibers signal pain. The encoding of itch and pain sensations by the same nerve fiber type via the distinct discharge patterns of primary afferents thus represents an interesting signaling mechanism that should be addressed in future studies, which should be also expanded to elderly persons and youth.

## Supplementary Information

**Additional file 1.** *Text 1 and Figure S1*: Information about the Bradykinin effect on itch and pain sensations evoked by beta-Alanine, Bam-8.22 and cowhage. *Figure S2*: Proportion of volunteers and number of volunteers perceiving an itch sensation with a NRS rating ≥ 1 during a 1-min sinusoidal stimulation before and after pruritogen application.

## Data Availability

The datasets used and/or analysed during the current study are available from the author on individual request.
