# Correction to: Peripheral signaling pathways contributing to non-histaminergic itch in humans

**DOI:** 10.1186/s12967-024-05941-x

**Published:** 2025-01-13

**Authors:** Andrea Fiebig, Victoria Leibl, David Oostendorf, Saskia Lukaschek, Jens Frömbgen, Maral Masoudi, Andreas E. Kremer, Marion Strupf, Peter Reeh, Miriam Düll, Barbara Namer

**Affiliations:** 1 Research Group Neuroscience, Interdisciplinary Centre for Clinical Research, Faculty of Medicine, RWTH Aachen University, Aachen, Germany; 2 Institute of Neurophysiology, Uniklinik RWTH Aachen University, Aachen, Germany; 3 Institute of Physiology and Pathophysiology, University of Erlangen-Nürnberg, 91054 Erlangen, Germany; 4 Department of Gastroenterology and Hepatology, University Hospital Zürich, University of Zürich, Zurich, Switzerland; 5 Department of Medicine 1, University Hospital Erlangen and Friedrich-Alexander-University Erlangen-Nürnberg, Erlangen, Germany

**Journal of Translational Medicine volume 21, Article number: 908 (2023)**

10.1186/s12967-023-04698-z

Following publication of the original article [1], we have been notified that Author contributions’ declaration was published incorrectly.

It is now as follows:

BN designed the study; BN and AF conducted the MNG experiments and analyzed the data; JF, VL, AF, MS, DO and MM conducted the psychophysics experiments and analyzed the data; AF created the figures and wrote the manuscript. PR, AK, MD and BN critically reviewed and finalized the manuscript. All authors approved the final version of the manuscript.

It should be as follows:

BN designed the study; BN and AF conducted the MNG experiments and analyzed the data; JF, VL, AF, MS, DO, SL and MM conducted the psychophysics experiments and analyzed the data; AF created the figures and wrote the manuscript. PR, AK, MD and BN critically reviewed and finalized the manuscript. All authors approved the final version of the manuscript.

The original article was updated.
