# Supplementary material for: Peripheral signaling pathways contributing to non-histaminergic itch in humans
Source: J Transl Med. 2023 Dec 12;21:908. doi: 10.1186/s12967-023-04698-z (PMC10717026; doi:10.1186/s12967-023-04698-z)
Supplement: Supplementary file 1 — Additional file 1. Text 1 and Figure S1: Information about the Bradykinin effect on itch and pain sensations evoked by beta-Alanine, Bam-8.22 and cowhage. Figure S2: Proportion of volunteers and number of volunteers perceiving an itch sensation with a NRS rating ≥ 1 during a 1-min sinusoidal stimulation before and after pruritogen application. [file 12967_2023_4698_MOESM1_ESM.docx]

**Additional file 1**

***Bradykinin***

Bradykinin had no significant effect on the itch or pain sensation evoked by β-alanine (Figure S1). The mean cumulative itch or pain ratings with pre-injection of the control solution were in a similar range (itch 16 ± 5.6; pain 10.13 ± 3.5) as with bradykinin (itch 14.88 ± 7.2; pain 8.13 ± 3.62). Maximum itch and pain ratings did not differ between control and bradykinin pre-injection treatments (control itch 1.45 ± 0.44, pain 1.1 ± 0.33; bradykinin itch 1.13 ± 0.44, pain 0.8 ± 0.33).

Bradykinin pre-injection significantly decreased itch and pain sensations evoked by BAM 8-22 (itch 15.5 ± 5.7 to 6.8 ± 3.5; Wilcoxon matched pairs, p = 0.04; pain 11.2 ± 4.86 to 2.7 ± 1.08; Wilcoxon matched pairs, p = 0.01). Bradykinin pre-injection reduced the maximum pain sensation (control 1.15 ± 0.46; bradykinin 0.5 ± 0.17; Wilcoxon matched pairs, p = 0.02) and itch sensation (control 1.35 ± 0.34; bradykinin 0.8 ± 0.26; Wilcoxon matched pairs, p = 0.10) in comparison to control (Figure S1).

The pre-injection of bradykinin caused significantly reduced the cumulative itch sensation caused by cowhage spicules (from 66.09 ± 12.3 to 47.03 ± 9.06; Wilcoxon matched pairs, p = 0.04) and also the cumulative pain ratings (from 17.16 ± 5.89 to 13.96 ± 4.4; Wilcoxon matched pairs, p = 0.01). The proportion of itch and pain with significantly higher maximum itch ratings was not changed by bradykinin (control conditions, cowhage itch 2.75 ± 0.52, pain 1.65 ± 0.52; Wilcoxon matched pairs, p = 0.04; bradykinin itch 2.43 ± 0.49, pain 1.23 ± 0.39; Wilcoxon matched pairs, p = 0.02).


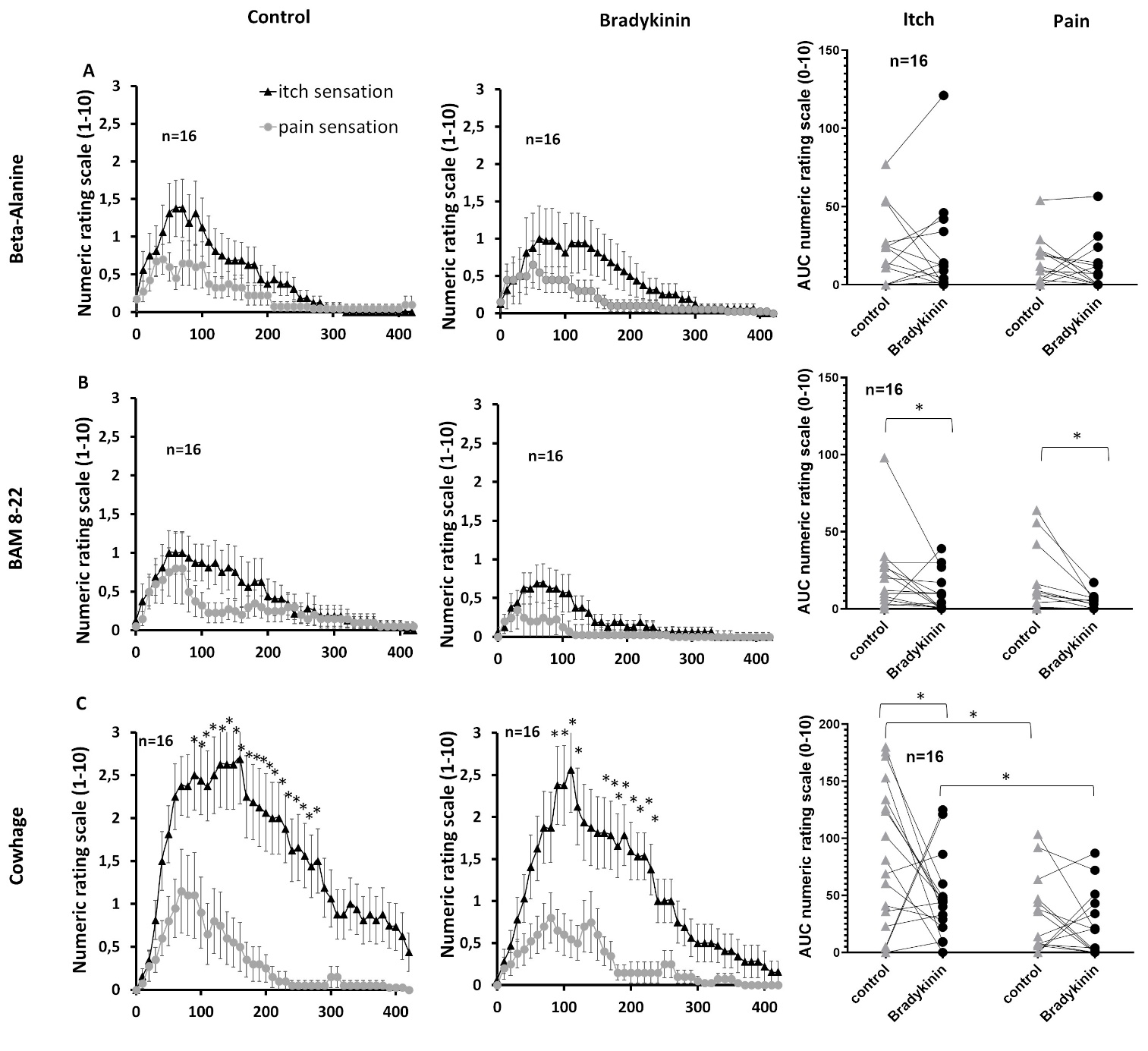


**Figure S1. Modulation of non-histaminergic itch by the inflammatory mediator bradykinin. The first column shows the numeric rating scale (values are means ± SEM) for 300 s after the injection of the pruritogen with the pre-injection of a control solution. The second column shows the same as the first, but with a pre-injection of bradykinin. The third column compares the effects of the pre-injection of bradykinin and the pre-injection of the control solution on the AUC of the numeric rating scale caused by the pruritogen injections. (A) Injection of β-alanine caused almost the same itch and pain sensation following pre-injection of bradykinin or pre-injection of the control solution. (B) The pre-injection of bradykinin caused a slight decrease in the pain and itch sensation after the BAM 8-22 injection. (C) The pre-injection of bradykinin caused a slightly lower itch sensation of the cowhage extract injection but did not affect the pain sensation.**


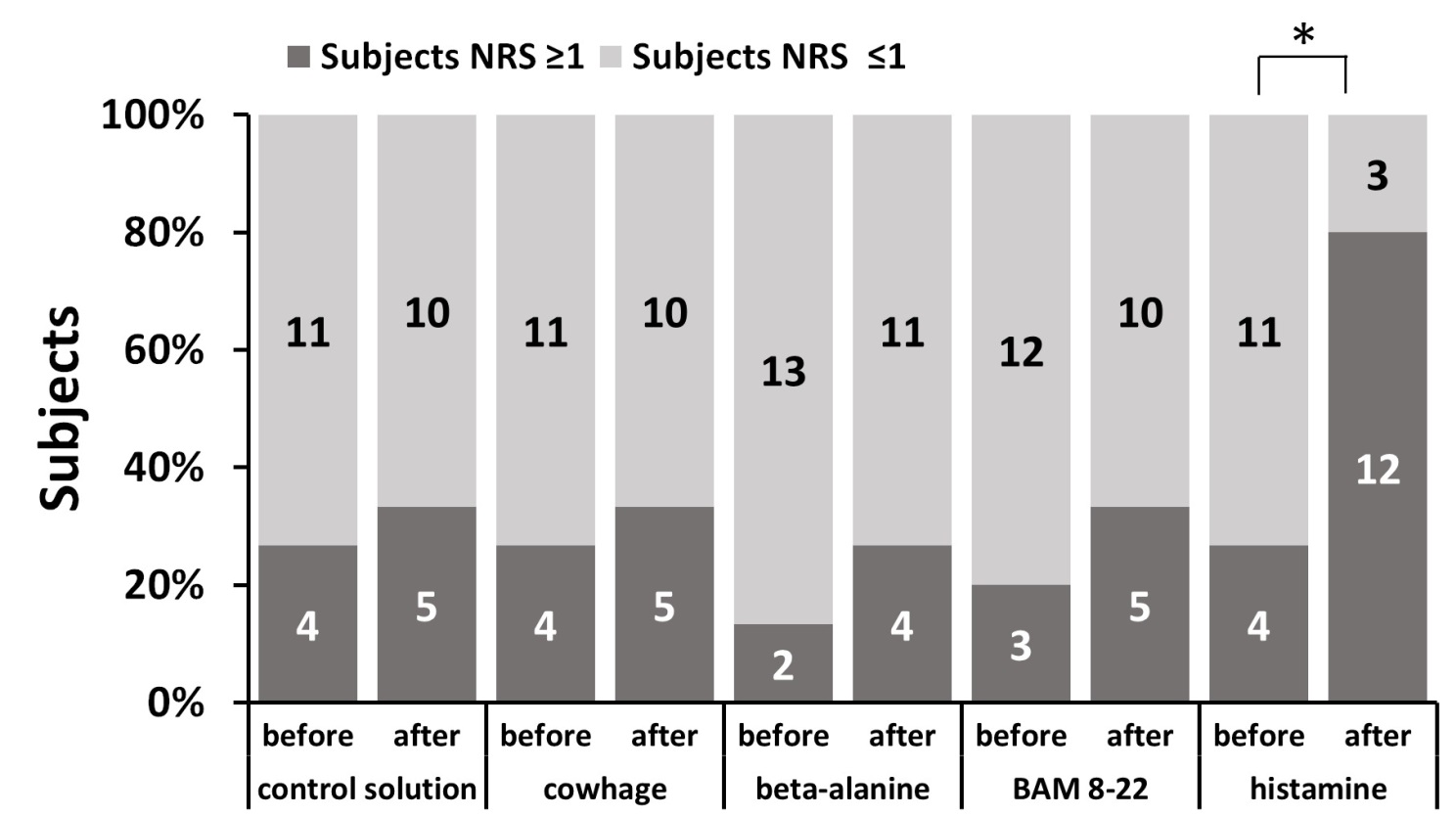


**Figure S2. Numbers of subjects perceiving an itch sensation with a NRS rating ≥ 1 during a 1‑min sinusoidal stimulation before and after pruritogen application. Number of subjects with NRS ≥ 1 and number of subjects with NRS ≤ 1 are shown as absolute numbers in stacked columns, while labeling on the y-axis includes the corresponding percentage.**
